# Heterogeneous Multilayer Nanopores via Chemically Tuned Dielectric Breakdown for Single-Molecule Sensing

**DOI:** 10.1002/smll.202513242

**Published:** 2026-03-06

**Authors:** Chaoming Gu, Kamruzzaman Joty, Navod Thyashan, Ivan Vlassiouk, Liam Collins, Xingye Zhang, Christopher T. Nelson, Nathan Taton, Sangyoup Lee, Min Jun Kim

**Affiliations:** 1 Department of Mechanical Engineering, Southern Methodist University, Dallas, Texas, USA; 2 Center For Nanophase Materials Sciences, Oak Ridge National Laboratory, Oak Ridge, Tennessee, USA; 3 College of Information Science and Electronic Engineering, Zhejiang University, Hangzhou, Zhejiang, China; 4 Department of Chemistry, Southern Methodist University, Dallas, Texas, USA; 5 Bionic Research Center, Biomedical Research Division, Korea Institute of Science and Technology, Seoul, Republic of Korea

**Keywords:** 2D materials, CT-CDB, multilayer structure, single-molecule biosensing, solid-state nanopore

## Abstract

Solid-state nanopores are powerful platforms for single-molecule sensing, yet their performance is often constrained by fabrication complexity, noise, and limited control over surface properties. Here we report a direct method to fabricate heterogeneous multilayer nanopores using chemically tuned controlled dielectric breakdown (CT-CDB). We integrate hBN, MoS_2_, or graphene atop a silicon nitride membrane to form five distinct bilayer and tri-layer architectures, with bare SiN_x_ nanopore as a control. CT-CDB achieves pore formation reproducibly through material-stacks with high efficiency, good pore size control, and strong yield, validated by various characterizations. Transferrin protein translocation experiments, supported by simulations, reveal that multilayer configurations modulate protein conformations, ionic current blockade and dwell time distributions, reflecting combined effects of membrane type, interfacial chemistry, and local electric field gradients. A supervised machine learning framework is implemented to assist identifying multilayer structure effects embedded in signal signatures, with over 96% accuracy. This work presents a modular and scalable framework for functional nanopore engineering with complex structural integration, thereby expanding the potential of 2D materials in single-molecule sensing applications.

## 1 | Introduction

Solid-state nanopores (SSNs) are emerging as powerful tools for label-free, single-molecule detection [1]. Advances in device geometry and materials [2–6], fabrication methods [7–10] and detection stratigies [11–14] have expanded their applications in biosensing. Despite these developments, nanopore fabrication remains a central challenge, particularly for complex or multilayered structures where precise geometry, stability, and surface chemistry should be controlled simultaneously.

Conventional *ex situ* methods, such as focused ion beam (FIB) or transmission electron microscopy (TEM) drilling, offer precise control over pore dimensions but are costly, low-throughput, and can damage sensitive materials [5, 15, 16]. In contrast, *in situ* dielectric breakdown (DB) techniques offer a scalable, cost-effective alternative for creating pores directly in solution, enabling robust operation and longer device lifetimes [10, 17–19]. Chemically tuned controlled dielectric breakdown (CT-CDB) further improves pore-size control and stability through regulation of electrolyte composition and oxidative conditions during breakdown [20].

Single-material nanopores, such as those made of SiN_x_, offer high mechanical and chemical stability [21], yet their thickness limits spatial resolution at the single-molecule level [22] and fast translocation [23, 24]. Atomically thin 2D materials like MoS_2_ and graphene provide improved resolution, but the performance is influenced by higher electrical noise [25], clogging [26] and poor robustness [27]. Integrating these 2D materials with conventional membranes represents one potential route to balancing the complementary strengths of both classes of materials.

In this work, we extend CT-CDB to fabricate heterogeneous multilayer nanopores by stacking 2D materials (hBN, MoS_2_, and graphene) on SiN_x_ membranes to form five bilayer and trilayer architectures, alongside bare SiN_x_ controls. These materials possess contrasting electrical and chemical properties: hBN is insulating and chemically inert, MoS_2_ is semiconducting, and graphene is conductive and hydrophobic; such that their integration creates heterointerfaces expected to influence local electric fields, ionic transport, and biomolecule-surface interactions. To investigate these effects, we used holo-human serum transferrin (holo-hSTf), a protein with compact tertiary structure, moderate size, and good electrophoretic mobility, as a model analyte [28]. Holo-hSTf is a physiologically relevant glycoprotein (~80 kDa) responsible for iron transport in blood circulation [29]. Its biomedical importance in iron metabolism and disease diagnostics makes it a valuable target for nanopore sensing studies. Unlike the widely used *λ*-DNA, which provides strong signals due to its size and uniform charge but lacks conformational variability, transferrin enables evaluation of nanopore sensitivity to more delicate protein-level differences. Translocation experiments revealed architecture-dependent variations in current blockade and dwell time distributions, suggesting roles for interfacial chemistry and dielectric environment in shaping protein passage. The performance of the heterogeneous multilayer nanopore fabrication by enhanced CT-CDB is compared with other conventional fabrication methods in Table 1

To further probe these complex signatures, we implemented supervised machine learning (ML) to analyze more than 300,000 events across the six pore architectures. ML was applied here as a complementary tool to identify multidimensional feature correlations that conventional histogram analysis may overlook. The framework achieved >96% classification accuracy across pore types, highlighting that subtle but reproducible structural fingerprints are embedded in the translocation signals.

While demonstrated here with a single protein and pore sizes of 20 ± 2 nm, the approach illustrates how integrating multilayer nanopore fabrication with advanced analytics can provide new insights into structure-signal relationships and enable future strategies for nanopore quality control and rational device design.

## 2 | Results and Discussion

### 2.1 | Structural Design and Nanopore Fabrication by CT-CDB

**FIGURE 1 |:**
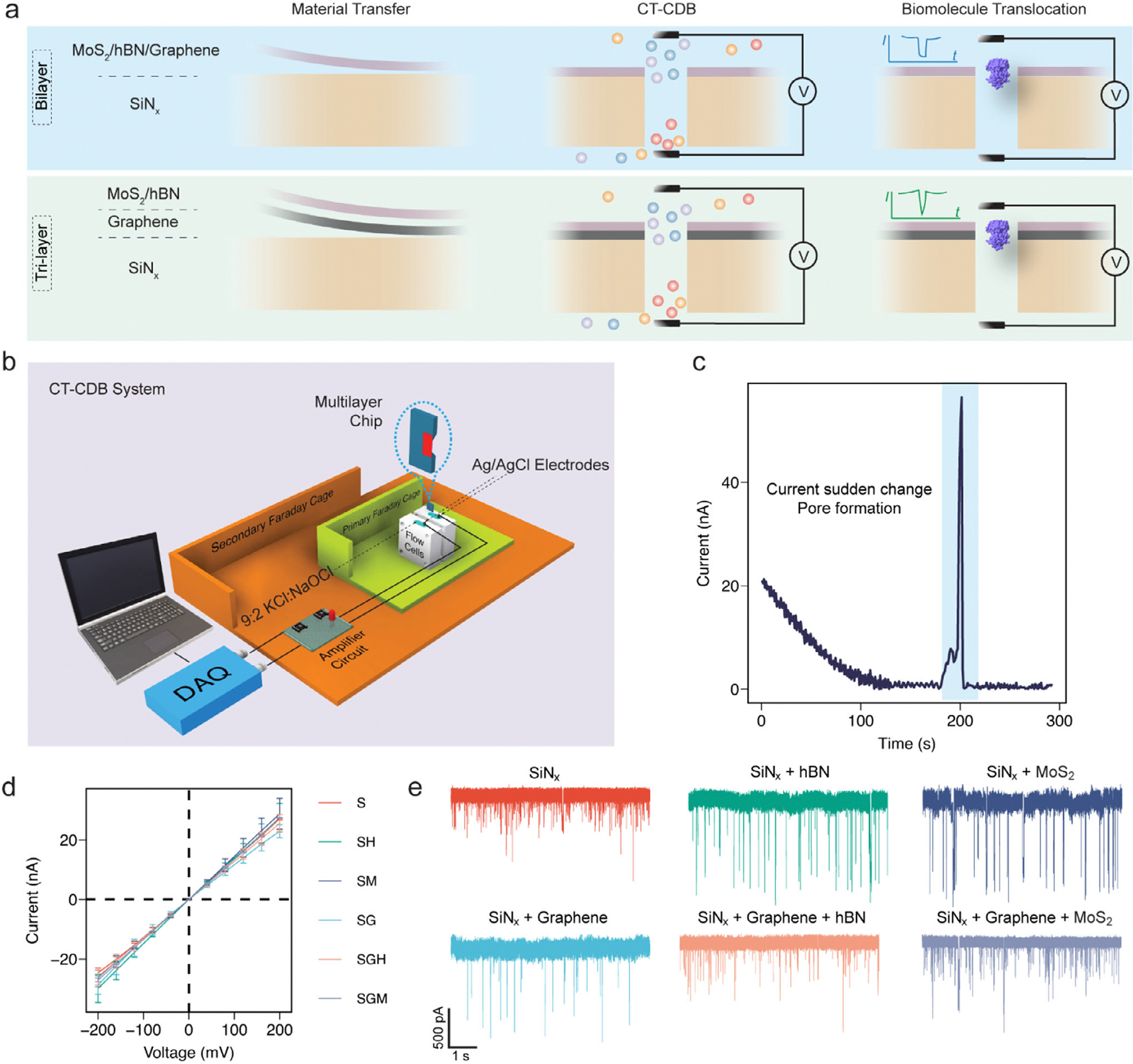
Fabrication of bilayer/tri-layer nanopores via CT-CDB and brief characterization. a. Three major steps for CT-CDB bilayer (top) and trilayer (bottom) nanopores: material transfer, CT-CDB, and biomolecule detections. Tri-layer materials are transferred one by one. Small balls in CT-CDB step with different colors are typical ions used: K^+^, Cl^−^, Na^+^ and OCl^−^. Protein molecules are driven by external voltage and ionic current blockades are recorded. b. Schematic of CT-CDB system, with double Faraday cages for noise screening and necessary amplifier circuit/DAQ for voltage control and current monitor. c. CT-CDB current trace corresponding to ~14 nm thick SH structure with an initial electric field of 0.7 V/nm. The current sudden change peak (blue region) at ~200 s indicates the formation of a pore. Applied transmembrane voltage controlled by LABVIEW program will stop instantly once the peak occurs. d. I-V curves from various multilayer structures. The pore sizes estimated from I-V are all close to 20±2 nm in diameter and the error margin at every voltage implies good consistency of the fabrication. e. Nine-second holo-hSTf translocation traces at 100 mV from different structures, with good capture rate and high signal-to-noise ratio.

Our designs include three bilayers: SiN_x_+MoS_2_ (SM), SiN_x_+hBN (SH), and SiN_x_+graphene (SG), and two tri-layers: SiN_x_+graphene+MoS_2_ (SGM) and SiN_x_+graphene+hBN (SGH) (Figure 1a; Figure S1). Multilayer structures are fabricated using standard dry transfer of exfoliated 2D materials onto free-standing 12 nm thick SiN_x_ membranes [4]. After overnight stabilization to strengthen the bonding between SiN_x_ and extra materials [33], CT-CDB is performed in a 1 M KCl-Tris/NaOCl electrolyte mix using our customized system illustrated in Figure 1b (see Method section).

CT-CDB combined with ethanol-deionized water wetting enhancement enables rapid pore formation (≤10 min) in multilayer nanopores, as shown in Figure 1c. Additional short voltage pulses are applied for fine tuning of the pore size, achieving a size control precision on the order of ~1 nm. Considering the size of holo-hSTf [28], typical nanopores utilized in this study have nominal effective diameters of 20 ± 2 nm, estimated from open-pore ionic conductance measurements (Figure 1d) [34]. Here, the reported diameter reflects the electrically active constriction governing ionic transport rather than an idealized cylindrical physical aperture.

Nanopores with smaller nominal effective diameters (~16 nm) were also fabricated; however, for holo-hSTf sensing, these pores frequently exhibited persistent clogging and unstable baseline recovery, which precluded reliable statistical analysis (Figure S2). Accordingly, nanopores with effective diameters of ~20 ± 2 nm were selected as an experimentally robust operational regime for the multilayer architectures. The fabrication process is efficient and reproducible across all five architectures, as demonstrated in Figure 1d, the Method section, and Figure S3.

Figure S3 illustrates the comparison of ~20 nm multilayer pore fabrication time among CT-CDB, CDB and TEM. CT-CDB method can fabricate a single/multilayer nanopore within 10 min. While from our trials, CDB needs slightly longer time to form the pore but with worse pore quality, consistent with related study [20]. TEM requires at least 30 min with unavoidable vacuum-pumping, window finding and focus adjusting time, meanwhile the surface of 2D materials could be harmed by electron beam [35].

### 2.2 | Structural and Electrical Characterizations

Unlike idealized cylindrical nanopores, CT-CDB-fabricated multilayer nanopores inherently exhibit geometric variability, including tapering, asymmetric edges, and layer-dependent constrictions. As a result, no single geometric parameter can fully describe the effective sensing region in heterogeneous multilayer membranes.

**FIGURE 2 |:**
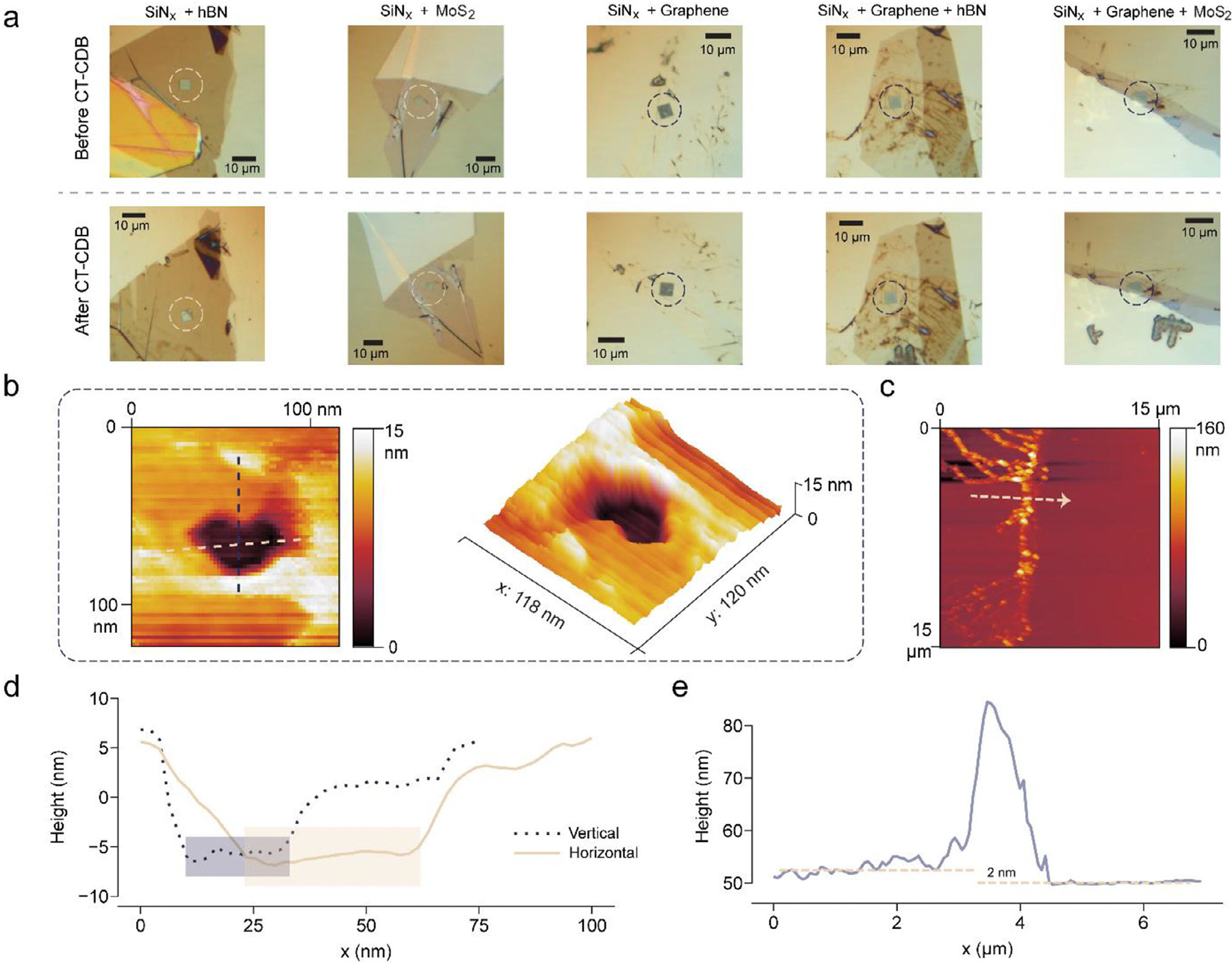
Validation and characterization of nanopore structures by optical microscope and AFM. a. Optical micrographs of five representative nanopore membrane architectures before (top) and after (bottom) CT-CDB. The membrane types include SiN_x_+hBN, SiN_x_+MoS_2_, SiN_x_+graphene, SiN_x_+graphene+hBN (SGH), and SiN_x_+graphene+MoS_2_ (SGM). All membranes are suspended over SiN_x_ window regions, marked by dashed circles. b. High-resolution atomic force microscopy (AFM) analysis of a nanopore fabricated in a tri-layer SGM membrane (SiN_x_+graphene+MoS_2_). Left: 2D height map revealing the localized breakdown site. Horizontal and vertical dashed lines indicate the axes used for cross-sectional profiling. Right: 3D view of the same region highlights pore morphology and confirms nanoscale depth. c. AFM scan across the interface between exfoliated hBN and the underlying SiN_x_. The dashed arrow marks the direction of the extracted height profile. d. Height profiles taken along the dashed lines in panel b. e. Line scan of the hBN/SiN_x_ edge in panel c, revealing a uniform step height of ~2 nm, consistent with 3–4 monolayers of hBN.

We verified pore dimensions mainly using open-pore ionic conductance fitting [34] and confirm structural integrity by optical inspection. Figure 2a shows the comparison of structure before and after CT-CDB pore fabrication. The colored circles mark out the SiN_x_ free-standing window (5 μm × 5 μm). On top of it, extra materials stay intact without position variance. Some thick parts far from the SiN_x_ window may fall off (Figure 2a SiN_x_+hBN) due to the fabrication and the environment phase-change for characterization, but this does not affect pore performance. Energy dispersive x-ray analysis (Figures S4 and S5) and Raman spectroscopy (Figure S6) were also applied to confirm the material types of different structures. The presence of translocation events for holo-hSTf sensing traces (Figure 1e) further verify the functionality and stability of the nanopores. These results demonstrate that CT-CDB enables reproducible nanopore fabrication across the multilayer architectures and thickness range investigated in this work.

AFM tapping-mode scans provide an intuitive visualization of CT-CDB-fabricated multilayer nanopores, although the technique does not yield a quantitatively precise determination of the effective pore size (Figure 2b). The nanopore exhibits a depressed central region consistent with material removal, surrounded by topographical ripples likely due to strain relaxation in the stacked layers. Horizontal and vertical dashed lines mark the axes used for cross-sectional profiling. The lateral dimensions of the nanopore are approximately 23 nm × 35 nm (Figure 2d), consistent in order of magnitude with conductance-derived estimates [36]. Certain degrees of enlargement and pore-shape distortion are attributed to environmental changes before and during measurement (liquid-air transitions) as well as possible tip convolution effects inherent in AFM [37], which do not necessarily reflect the effective pore constriction governing ionic transport. The HAADF-STEM scans were taken for the entire membrane window at 2 nm/pixel resolution and black levels calibrated ensure hole/vacuum identification (Figure S7). However, due to the structure complexity and liquid-air phase transitions during characterization, both TEM and AFM imaging exhibit pronounced nanoscale surface contamination on the chips (Figures S7 and S8), which makes direct and precise visualization of the nanopore constriction challenging. These limitations underscore the need to interpret pore dimensions in terms of their effective electrical constriction. A comprehensive discussion of this interpretation is provided in Supplementary Note on Interpretation of Pore Size and Structural Variability in Multilayer Nanopores.

The effective pore length was estimated through AFM-based height profiling of the additional 2D material layers. The measured thicknesses of hBN, MoS_2_, and graphene were approximately 2 nm (Figure 2c,e), 3 nm (Figure S9a,c), and 2 nm (Figure S9b,d), respectively. The full depth of the depression is ~14 nm (Figure 2d), consistent with breakdown through a multilayered stack comprising ~2 nm of 2D material and 12 nm of SiN_x_. This suggests that CT-CDB creates through-pores that span the entire membrane thickness while preserving structural layering. According to the measurements, the resulting effective pore lengths are approximately 14 nm for bilayer configurations and 16 nm for tri-layer structures.

In solid-state nanopores, the absolute current noise power spectrum scales with the DC ionic current [38], making direct comparisons across devices with different pore size or conductance ambiguous. To address this, we employed a Hooge-type normalization *C*_1/*f*_, in which the PSD is divided by the squared DC current and fitted to a 1/*f*^*β*^ dependence [39]. This dimensionless coefficient, analogous to the Hooge parameter in semiconductors, reflects the intrinsic fluctuation of ionic conductance at the nanopore-electrolyte interface and enables a meaningful comparison of 1/f noise levels across different pores and material systems. Figures S10 and S11 reveals that nanopores with hBN layer on top suppress low-frequency 1/f noise across various voltages compared to bare SiNx, while MoS_2_ and graphene layers increase noise levels to different degrees [22, 40, 41]. This suggests the insulation of hBN aids noise suppression. For tri-layer ones, the existence of graphene increases the noise but hBN layer on top makes it lower than SG structure due to longer pore length [31] and hBN’s own noise-suppressing feature for SGH.

### 2.3 | Electrokinetic Analysis of Bilayer Nanopores for Holo-hSTf Sensing

Multilayer nanopores fabricated by CT-CDB inherently exhibit geometric asymmetry, tapering, and pore-to-pore variability, particularly in heterogeneous membrane architectures. These structural features influence access resistance, local electric-field distributions, and capture-orientation bias, thereby contributing to event-level variability in ionic conductance and dwell-time statistics. At the single-event level, protein translocation through solid-state nanopores is intrinsically stochastic, especially for globular proteins such as holo-transferrin, due to differences in capture orientation, transient conformational fluctuations, and short-lived protein-surface interactions. In multilayer nanopores, this intrinsic variability is further amplified by layer-dependent interfacial effects and spatially varing electric-field gradients. Accordingly, the translocation analyses presented in this work emphasize statistically reproducible ensemble trends extracted from large datasets collected across multiple conductance-matched nanopores, rather than relying on individual events or idealized pore geometries.

**FIGURE 3 |:**
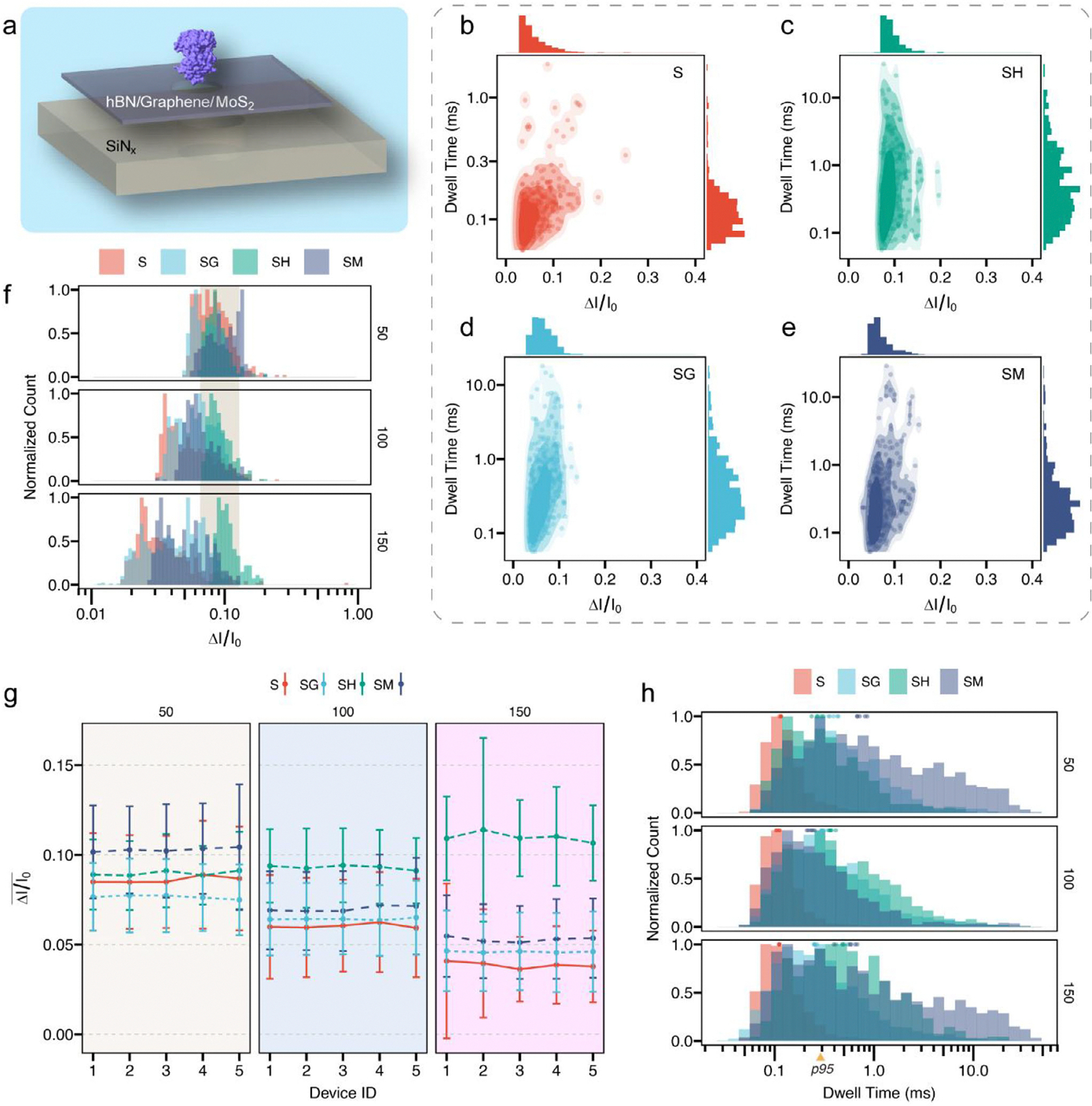
Statistical analysis on holo-hSTf translocation from bilayer structures. a. Schematic of three types of bilayer nanopore structures. b. Scattered distribution of transferrin translocation events from SiN_x_ (marked as S) nanopore between relative current ΔI/I_0_ and dwell time at 100 mV. The corresponding histogram distributions are on top and right sides. c. Scattered distribution of transferrin translocation events from SH nanopore at 100 mV. d. Scattered distribution of transferrin translocation events from SG nanopore at 100 mV. e. Scattered distribution of transferrin translocation events from SM nanopore at 100 mV. Compared with S, hSTf translocations from bilayer nanopores show wider dwell time distribution and narrower ΔI/I_0_ distribution. f. ΔI/I_0_ normalized histogram distribution from different bilayer structures and SiN_x_ nanopore, across 50, 100, and 150 mV. Colored strip marks the peak locations from SH nanopore. The peak stability at different voltages indicates the ability of preserving hSTf conformation from SH structure. g. Cross-device mean ΔI/I_0_ comparison at various voltages. The variation trend remains consistent among different devices with the same structure type. h. Dwell time normalized histogram distribution from different bilayer structures and SiN_x_ nanopore, across 50, 100, and 150 mV. The location of p95 point of SiN_x_ nanopore implies wider dwell time distribution of bilayer structures. Points over histogram demonstrate dwell time medians from other devices with the same structure type. The consistent trend also shows the robustness and reliability of bilayer nanopores fabricated by CT-CDB. Despite the apparent dispersion of individual translocation events, consistent ensemble-level trends are observed across multiple conductance-matched nanopores, supporting the robustness of the conclusions.

Holo-hSTf translocation events from bilayer structures (Figure 3a) were recorded at 50, 100 and 150 mV (see translocation traces in Figure 1e and Figure S12). Figure 3b–e and Figure S13 demonstrate the scattered distribution of relative current blockade (ΔI/I_0_) vs dwell time (ms) from SH, SM and SG structures compared with SiN_x_ (S). With extra 2D thin layer, the event distribution modes of bilayer nanopores are distinct from that of SiN_x_. Specifically, the cluster of ΔI/I_0_ from bilayer nanopores is concentrated more to right, while in SiN_x_, the distribution peak is closer to the lower left. Bilayer structures exhibit a broader dwell time distribution than SiN_x_, and a considerable portion of events are longer than 1 ms.

To inspect them more closely, the normalized histogram distribution of ΔI/I_0_ is demonstrated in Figure 3f. Across all the nanopores with similar pore size and pore length, a specific analyte (holo-hSTf in our case) is supposed to exhibit similar ΔI/I_0_ [42]. Therefore, the difference in ΔI/I_0_ (Figure 3f) primarily reflects the structures themselves at a given applied voltage. For SH structures, the ΔI/I_0_ distributions remain relatively stable across voltages (Figure 3f), which is consistent with the weak interaction between hBN and proteins reported in prior studies [43]. Unlike MoS_2_ and graphene [4, 44], minimal protein adhesion or deformation is expected in SH structures, which may help maintain holo-hSTf in a near-native transport state even under high bias. This is reflected in the consistent distribution peaks without positional shifts at different voltages (Figure 3f, colored strip). The calculated ΔI/I_0_ (0.06) by volume exclusion model [42] (See Supplementary Information, Volume Exclusion Model Note) further supports this effect. For other structures, different degrees of adhesion between protein and material surface may cause the state change of protein molecules, which decreases ΔI/I_0_ [45, 46]. Also as voltage increases, ΔI/I_0_ tends to decrease due to the minor deformation affected by higher electric field [47].

This phenomenon exhibits consistency across different samples, illustrated in Figure 3g. For each structure, at least 5 chips are tested. The average ΔI/I_0_ decreases with higher voltage except for SH type. This effect is more obvious at 150 mV, in which the mean values of ΔI/I_0_ of SH are more than two times higher than the rest. This consistency on the variation of ΔI/I_0_ not only proves the molecule preservation of hBN but provides further evidence confirming the stability and reproducibility of CT-CDB on multilayer nanopore fabrication.

As shown in Figure 3h, events from SH, SM, and SG structures all have dwell time distribution longer than 0.3 ms (p95 points of SiN_x_, meaning 95% events have dwell time shorter than 0.3 ms). Specifically, the percentage of > p95 events is 58% (SH), 51% (SG) and 50% (SM). The increase of pore thickness from ~12 to ~14 nm can only account for an estimated ~20–30% increase in dwell time according to established electrostatic and access resistance models [38, 48]. The much larger increase observed in our bilayer devices therefore cannot be attributed to pore length alone. This observation indicates that additional factors associated with the introduction of 2D layers contribute significantly to the observed transport slowdown. These materials are known to modify surface charge distributions or molecule-wall interactions, which substantially reduce the effective mobility of proteins and increase their residence times within the nanopore [49–51]. For SH nanopores, the longer dwell time of proteins is not attributed to strong direct interactions between hBN and the biomolecules, since hBN is known to exhibit relatively weak interactions with proteins [43, 52]. Instead, the effect is consistent with changes in the pore electrostatics environment. The presence of an hBN layer alters the local electric field distribution across the bilayer stack, which may reduce the electrophoretic driving force acting on the translocating protein. For SM and SG structures, stronger interaction between protein and 2D materials play the major role. COMSOL simulations based on the Poisson-Nernst-Planck equation (Table S1 and Figure S22a–c, and the Supplementary COMSOL Simulation Note for detailed design, boundary conditions, and simulation results) indicate SH, SM and SG exhibit potential changes of varying extents within the pore region. It should be noted that SH has smaller potential gradient compared to other bilayer structures, especially at the edge of the extra layer. Quantitively, at 100 mV, the potential difference inside the SH pore is ~7 mV lower than SM and SG structures, consistent with the prolonged translocation dwell time phenomenon in SH. For SM and SG structures, the interaction with proteins slows the translocation, but relatively stronger electrophoretic force counteracts part of the effect.

The colored dots above dwell time histograms (Figure 3h) correspond to the medians of dwell time distributions across devices. Despite minor deviations, the trend that bilayer structures prolong proteins’ translocation time keeps consistent, which again verifies the robustness and cross-device agreement of CT-CDB method on multilayer nanopore fabrication.

Figure S14 depicts capture rate of holo-hSTf in bilayer structures. Due to the surface hydrophobicity and noise [53], SG nanopores have the lowest capture rate. The average capture rates of other structures are more than 5 s^−1^. SH and SM nanopores even exhibit more than 15 s^−1^ at 150 mV with very few clogging, higher than bare SiN_x_ nanopores. This trend is consistent with enhanced electric-field focusing near the pore entrance induced by the introduction of the 2D layer, as suggested by the simulations shown in Figure S22. High capture rate also surpasses pure 2D nanopores (e.g., 0.7 s^−1^ for MoS_2_) [22], indicating better fabrication quality of CT-CDB and good performance of bilayer structure on protein sensing, even with the existence of 2D materials.

Overall, the distinct translocation behaviors observed in SH, SM, and SG nanopores reflect a coupled interplay between pore geometry, electrostatic environment, and material-dependent interfacial effects. Variations in ΔI/I_0_ are primarily sensitive to changes in protein-surface interactions, while dwell time distributions are influenced by the effective electrophoretic driving force shaped by local electric-field redistribution. These mechanisms are not mutually exclusive, and the observed signals likely represent a convolution of multiple transport and interaction processes rather than a single dominant factor.

### 2.4 | Electrokinetic and Comparative Analysis of Tri-Layer Nanopores for Holo-hSTf Sensing

**FIGURE 4 |:**
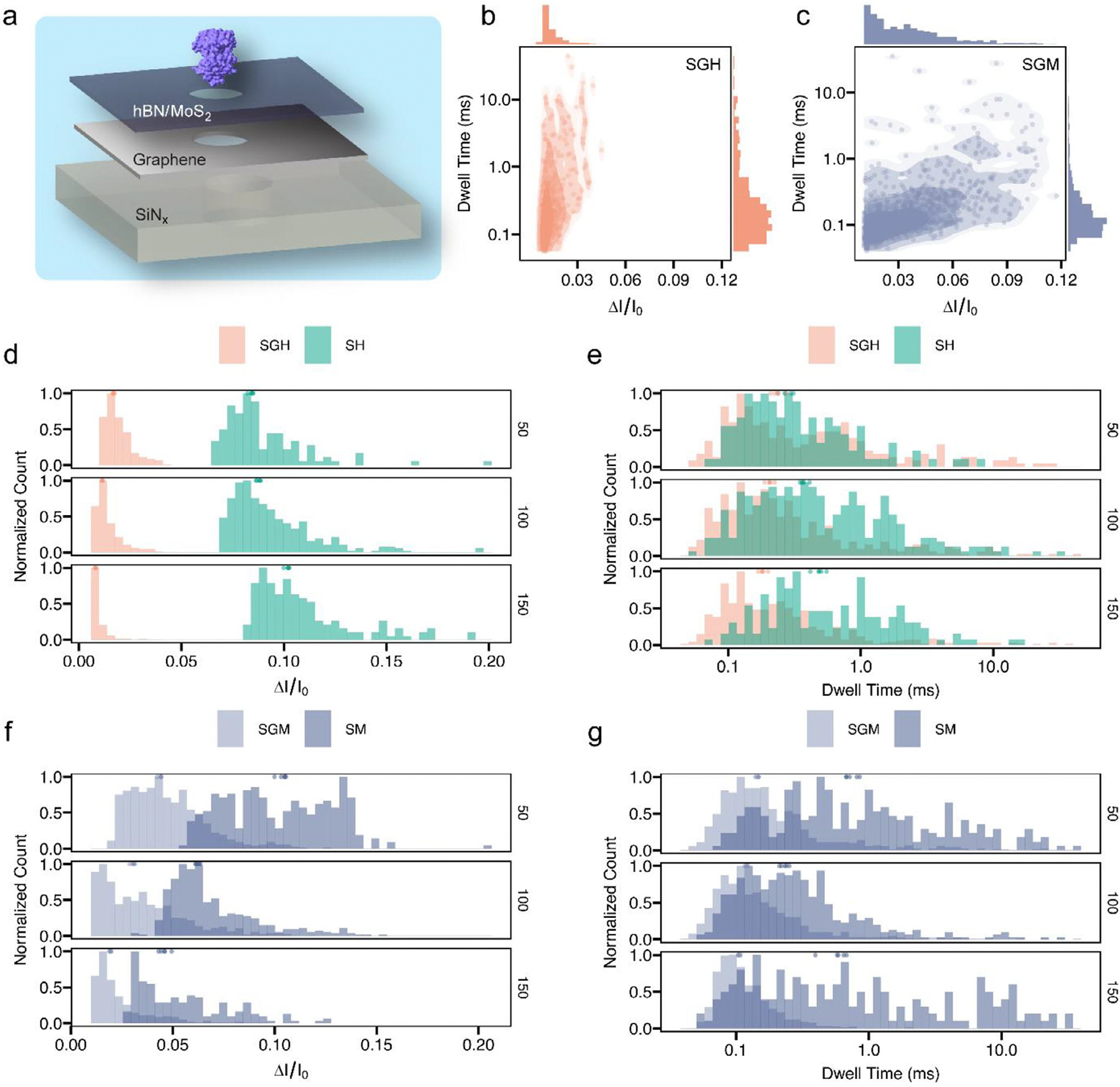
Statistical and comparative analysis on holo-hSTf translocation from tri-layer structures. a. Schematic of two types of tri-layer nanopore structures. b. Scattered distribution of hSTf translocation events from SGH nanopore at 100 mV, between relative current ΔI/I_0_ and dwell time. The corresponding histogram distributions are on top and right sides. c. Scattered distribution of hSTf translocation events from SGM nanopore at 100 mV, between relative current ΔI/I_0_ and dwell time. d. Comparative normalized histogram of ΔI/I_0_ between SGH and SH structures across 50, 100 and 150 mV. At all voltages, the peaks and the distributions from two structures are clearly separate. e. Comparative normalized histogram of dwell time between SGH and SH. The range of dwell time is similar but some of the long events in SGH nanopore are suppressed. f. Comparative normalized histogram of ΔI/I_0_ between SGM and SM structures across 50, 100 and 150 mV. There are peak shifts from SGM, but part of the signals remains overlapped with SM nanopore. g. Comparative normalized histogram of dwell time between SGM and SM. The long events from SGM nanopore are significantly suppressed compared with SM nanopore. Despite the dispersion of individual events, consistent shifts in ensemble distributions and voltage-dependent trends are observed across conductance-matched nanopores, supporting the robustness of the reported conclusions.

Tri-layer nanopores (Figure 4a) integrate graphene as the middle layer and hBN/MoS_2_ as the top layer. Slightly thicker structures improve mechanical strength, while graphene, as a semi-conductive layer, can modulate the local electric field inside nanopore and make the structure extensible for in-plane current sensing [54]. CT-CDB further enables reliable fabrication of these heterogeneous multilayer architectures without requiring additional alignment or drilling steps.

The scattered distributions (Figure 4b,c; Figure S15) show very distinct modes between SGH and SGM. For SGH, ΔI/I_0_ cluster is much narrower and smaller than SGM. This difference is more obvious between SH and SGH structures (Figure 4d; Figure S16). With the existence of graphene 2D layer in the middle, at all applied voltages, SGH nanopores exhibit the lowest relative current blockade values (~0.01–0.02) despite similar pore diameters (~20 ± 2 nm) and effective thicknesses (~16 nm). This consistent signal attenuation likely stems from the electrostatic environment imposed by the multilayer structures. The combination of a dielectric hBN layer and an in-plane conductive graphene interface modulates the electric field distribution and screening behavior across the nanopore [55], reducing the magnitude of ionic current perturbation during protein translocation. Furthermore, hBN’s inert surface chemistry may limit proteinpore interaction [56], leading to less obstructive translocations and lower signal amplitudes.

SGH has more signals with longer dwell time than SGM across different devices and voltages (Figure 4b,c; Figure S17). COMSOL simulations (Figure S22e) corroborate a reduced field gradient and electrophoretic force in SGH (~7 mV lower than SGM), consistent with our experimental findings on slow-down effect. Compared with SH structure (Figure 4e; Figure S18), the dwell time distribution ranges are similar, but the proportion of longer events from SGH is lower. Inserting a conductive graphene layer into the SiN_x_-hBN stack reduces the effective pore length because the graphene acts as an equipotential boundary that interrupts the continuous potential drop across the dielectric. This structural division splits the pore into two shorter segments and concentrates the electric field near the graphene interface [57, 58], thereby accelerating transferrin transport and yielding shorter dwell times than in SH pores of comparable physical thickness.

For SGM structure, the reduction in current blockade is less pronounced and the distributions partly overlap with SM structure (Figure 4f; Figure S19), consistent with the residual MoS_2_-protein interactions that sustain higher blockade amplitudes [59]. However, the dwell time statistics show a more striking change: while the mean translocation times decrease for both SGH and SGM, events with longer dwell time observed in SM are suppressed in SGM (Figure 4g; Figure S20). This suggests that graphene not only sharpens the electric field but also screens MoS_2_ surface affinity, thereby reducing transient protein trapping and narrowing the dwell time distribution [57, 58].

Protein capture rates of tri-layer nanopores (Figure S21) are similar to those of SiN_x_ nanopores. While the average capture rates increase modestly with applied bias, all variations fall within experimental uncertainty, indicating no measurable degradation in capture efficiency. This behavior reflects the high quality of the nanopores and the reliable fabrication of multilayer nanopores enabled by CT-CDB. Compared with previously reported TEM-drilled graphene-MoS_2_ nanopores with a capture rate of 0.34 s^−1^ [4], the tri-layer structures achieve more than an order-of-magnitude higher sensing throughput.

Overall, signal formation in tri-layer nanopores is governed by a coupled interplay between electrostatic modulation and interfacial effects introduced by the multilayer architecture. The insertion of graphene acts as an equipotential boundary that redistributes the electric field and shortens the effective electrophoretic length of the pore, enabling concurrent suppression of current blockade and acceleration of protein translocation relative to purely dielectric stacks. Variations in ΔI/I_0_ and dwell time across different tri-layer configurations follow the same electrostatic and interfacial principles identified in bilayer nanopores, indicating that transport behavior is not dictated by pore geometry alone. These results highlight the utility of multilayer stacking as a modular strategy capable of tuning signal blockade and translocation dynamics without compromising capture efficiency.

### 2.5 | Machine Learning for Event Signatures Analysis From Different Structures

Solid-state nanopore sensors generate rich electrical signatures from biomolecule translocation yet extracting reproducible structural insights from these noisy and heterogeneous signals remains a major challenge. Conventional histogram-based analysis often struggles to capture subtle yet systematic differences in event features that may reflect the characteristics of membrane architectures or surface properties. To address this, we developed a supervised machine-learning (ML) framework as a proof-of-concept analytical tool for decoding nanopore-dependent signatures from protein translocation data. Machine learning methods have shown remarkable promise in nanopore analytics, providing label-free discrimination and identification of biomolecules by utilizing multidimensional event features rather than single-parameter thresholds [60–62]. Here, we extended this concept to test whether extracted and engineered features from holo-hSTf translocations could encode the physical characteristics of the underlying nanopore membrane. Each event was described by feature vectors such as dwell time, current blockade, relative blockade, translocation velocity, signal-to-noise ratio, event asymmetry, and many more (Figure S24 and Table S2).

**FIGURE 5 |:**
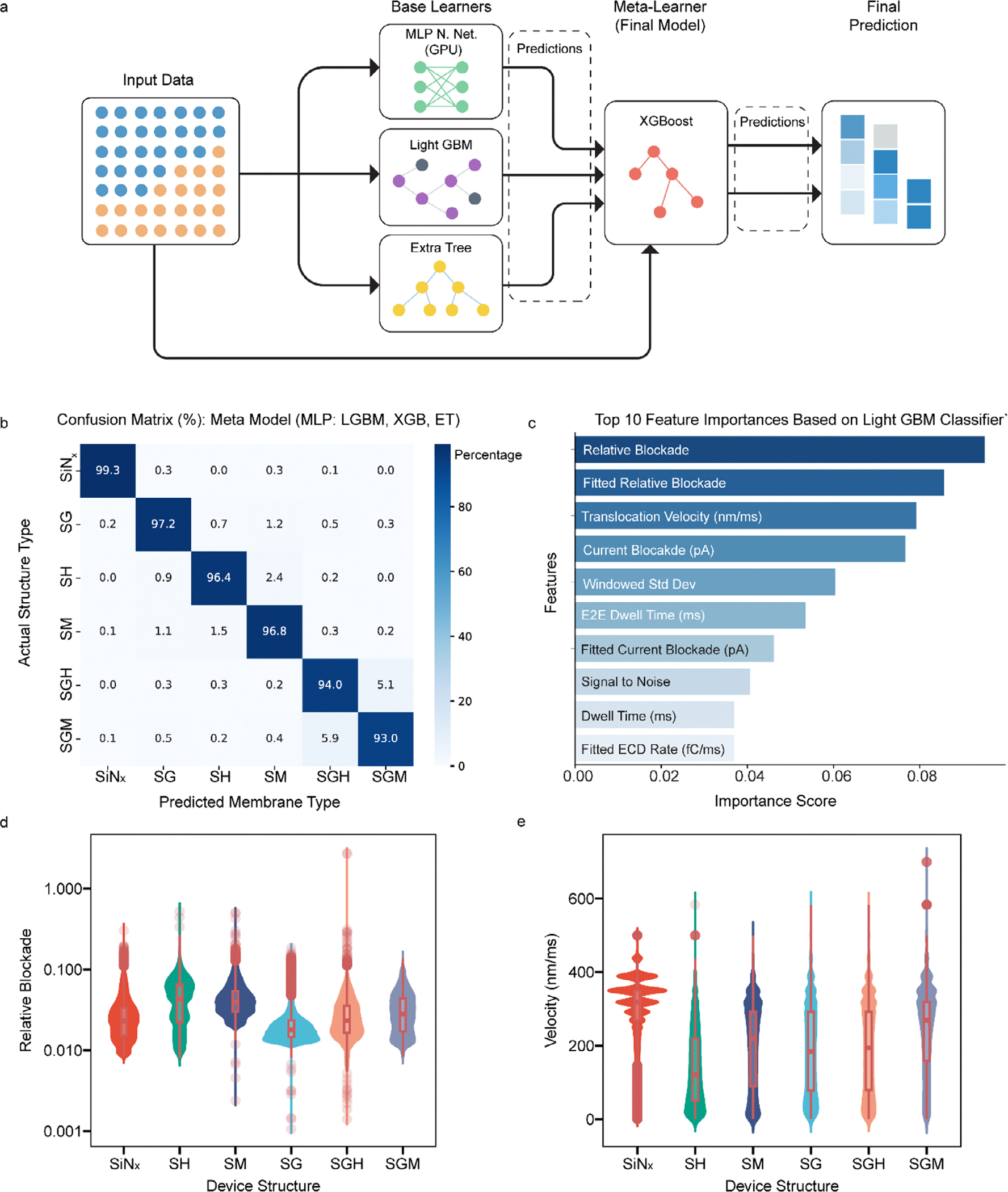
Machine learning classification of structure types based on translocation features. a. Schematic of the stacked ensemble learning architecture. Three base learners: a multilayer perceptron (MLP), a gradient-boosted tree model, and an extremely randomized trees model, generate independent predictions, which are passed to a meta-learner to yield the final classification output. b. Confusion matrix showing the classification performance of the final stacked model on an independent test dataset. The model achieved an overall accuracy of 96.33% across six membrane types: bare SiN_x_, SiN+Graphene (SG), SiN+MoS_2_ (SM), SiN+h-BN (SH), SiN+Graphene+h-BN (SGH), and SiN+Graphene+MoS_2_ (SGM). Misclassifications were most frequent between SGH and SGM membranes, whereas SiNx and SH types exhibited the highest separability. c. Top 10 most informative features used in classification, ranked by their relative importance. High-ranking features included relative current blockade, translocation velocity, dwell time, signal-to-noise ratio, and fitted electric charge displacement (ECD) rate, among others. d. Violin plot showing the distribution of relative current blockade (ΔI/I_0_) for each membrane type. Variations in distribution shape and median reflect the impact of membrane composition on protein–nanopore interaction. e. Violin plot of translocation velocity (nm/ms) across membrane types, revealing that multilayered configurations (SGH, SGM) generally exhibit slower and more confined translocation dynamics compared to bare SiN_x_.

Our dataset comprised over 300,000 events recorded across six nanopore architectures: SiN_x_, SH, SM, SG, SGH and SGM. To reduce bias from pore-to-pore variability, events were stratified and balanced into training and testing subsets (Table S3). We trained a stacked ensemble learning model combining three complementary base learners: a multilayer perceptron (MLP), Light gradient boosting machine (LightGBM), and extremely randomized trees (ExtraTrees), with an eXtreme Gradient Boosting (XGBoost) meta-learner (Figure 5a) [63–65]. Stacked ensemble architectures exploit complementary strengths of heterogeneous base classifiers, improving classification accuracy and generalizability compared to single-model approaches [66]. The final model configuration was carefully selected after extensive benchmarking against alternative stacking arrangements (Figures S25–S27). The optimized ensemble model achieved a classification accuracy of 96.33% (Figure 5b), with strong discriminability across all membrane types. Bare SiN_x_ and SH membranes exhibited the highest separability (Figure S29), while moderate overlap was observed between composite architectures such as SGH and SGM, likely due to their shared graphene interface. Validation on an independent SiN_x_ dataset yielded comparable accuracy (Figure S28), supporting model generalizability.

To interpret the basis of classification, we ranked the ten most informative features (Figure 5c). Dominant discriminative features included relative current blockade, translocation velocity, dwell time, fitted electric charge deficit (ECD) rate, and signal-to-noise ratio, highlighting both geometrical and dynamical event characteristics as critical determinants for nanopore classification [66]. SHAP analysis confirmed the physical relevance of these metrics across membrane types (Figure S31), aligning with prior nanopore ML studies that emphasized the importance of combining geometric and kinetic descriptors [67]. These results indicate that even subtle differences in translocation dynamics contain quantifiable signatures of membrane architecture.

Analysis of relative current blockade and translocation velocity distributions (Figure 5d,e) revealed that multilayer configurations broadened and diversified the range of translocation behaviors compared to bare SiN_x_. Bilayer and multilayer configurations, particularly those incorporating hBN as top layer or graphene as middle layer, broaden and diversify the range of translocation behaviors, resulting in wider or more complex distribution shapes for both blockade and velocity. This effect arises from the interplay between the surface chemistry, dielectric properties, and thickness of the constituent layers. For instance, hBN is chemically inert and electrically insulating, which minimizes protein adsorption and electrostatic trapping, thereby stabilizing the current blockade against voltage fluctuations and promoting the preservation of protein conformation during passage [43, 56, 68]. Graphene, by contrast, is atomically thin and highly conductive, providing a hydrophobic and low-friction interface that can reduce protein-surface interaction and increase variability in the translocation path [4, 69]. When these materials are integrated into multilayer heterostructures, the resulting spatially heterogeneous electric fields and local potential gradients further modulate ion transport and protein movement, manifesting as both signal attenuation and enhanced diversity in translocation event characteristics [67, 68, 70, 71]. These findings are consistent with our earlier results showing voltage-independent blockade stabilization in hBN-rich pores and significant reduction of relative current blockade in multilayer stacks.

This framework demonstrates that ML can extract latent information about nanopore structure embedded within single-molecule ionic current traces. The present study was limited to holo-hSTf and ~20 nm pores, but the approach could be extended to other biomolecules and device geometries in future work. While the dominant descriptors identified by the ML analysis, such as relative blockade depth, dwell time, translocation velocity, and signal-to-noise ratio, are well established in nanopore sensing, the strength of the ML framework lies in its ability to quantitatively integrate these descriptors and reveal how their combined variations encode membrane architecture. The ML model is therefore used as a multivariate validation and comparison tool rather than as a mechanistic substitute for physical interpretation. Importantly, such classification is not intended to replace experimental knowledge of pore type, but rather to provide automated quality control in large-scale nanopore arrays where device variability can obscure material effects, as well as a new perspective for discovering structure-signal correlations that traditional analyses may overlook. This integration of ML with nanopore experiments offers a route toward more systematic characterization of heterogeneous devices and, ultimately, toward the optimization of nanopore platforms for biosensing and diagnostic applications.

## 3 | Conclusion

In this work, we establish a scalable and robust fabrication strategy for heterogeneous multilayer solid-state nanopores based on chemically tuned controlled dielectric breakdown (CT-CDB). By integrating SiN_x_ with representative 2D materials, including hBN, MoS_2_, and graphene, we realize bilayer and tri-layer nanopore architectures with controlled pore sizes (~20 nm) suitable for protein translocation studies. Electrical characterization confirms reproducible single-pore formation across multiple material stacks and membrane thickness, demonstrating the reliability of CT-CDB for constructing complex multilayer nanopores, despite current limitations in direct pore visualization using TEM and AFM.

Using holo-hSTf as a benchmark analyte, we show that multilayer nanopore architectures systematically modulate protein translocation behavior through a coupled interplay of electrostatic field redistribution and material-dependent interfacial interactions. Supported by COMSOL simulations, our results demonstrate that variations in current blockade depth and dwell time distributions arise from structural and dielectric effects intrinsic to the multilayer design, rather than from pore geometry alone. These findings establish multilayer stacking as an effective strategy for tuning translocation dynamics and signal characteristics in solid-state nanopores.

Beyond conventional statistical analysis, we further demonstrate that translocation signals encode rich and reproducible structural information specific to each membrane architecture. By leveraging a stacked ensemble machine learning framework, we achieve classification accuracies exceeding 96% across six distinct nanopore types. Importantly, this framework does not replace physical interpretation, but instead provides a quantitative, multivariate validation of structure-signal relationships and enables automated structural fingerprinting and quality control across large datasets and multiple devices.

This study defines CT-CDB as a flexible and scalable platform for rational multilayer nanopore engineering within the material combination, thickness range, and fabrication conditions explored here. The ability to integrate diverse 2D materials into electrically robust nanopores without additional alignment or drilling steps opens new opportunities for modular design and functional tuning. More broadly, the combination of multilayer nanopore architectures with data-driven signal analysis provides a general pathway toward next-generation nanopore biosensors with enhanced functionality, throughput, and adaptability for advanced single-molecule sensing and diagnostic applications.

## 4 | Experimental Section/Methods

### 4.1 | Material Acquisition and Transfer on SiN_x_ Substrate

To prepare multilayer structures, we mainly use mechanical exfoliation to get thin films of hBN (2Dsemiconductors USA), MoS_2_ (2Dsemiconductors USA) and graphene (SixCarbon Technology) from the bulk materials. Standard dry transfer method [18] is applied for material transfer on free-standing SiN_x_ window chip (Norcada Inc). The free-standing area is 5 μm × 5 μm. For bilayer, we only transfer once; for multilayer, we first transfer one graphene film on the chip, then we get another layer of hBN/MoS_2_ on top of the graphene. For both cases, the SiN_x_ window part should be totally covered by all the materials. To strengthen the contact between substrate and transferred materials, the chip should be put still in a dry environment for at least 12 h, decreasing the possibility of material fall-off during the fabrication.

### 4.2 | Device Assembly, Preprocess, and CT-CDB Fabrication

The nanopore device consists of three parts: the chip with multilayer structure, the PDMS stamps for sealing, and PTFE customized flow cells. In the middle of PDMS stamps, a hole with ~2.5 mm in diameter is created by biopsy punch (TED PELLA Inc). Ethanol and deionized water (DI water) are used to clean PDMS stamps and flow cells. Two PDMS stamps clip the chip in the middle, and the holes should be aligned with the window area. This sandwich-like part is also aligned with the small channel on the flow cell, and we use four screws and nuts to make the whole assembly tight. By doing so, we can ensure that the solution won’t flow elsewhere but the nanopore channel. The final device is illustrated in Figure 1b.

1:1 (volume) ethanol and DI water mixture is added to flow cell reservoirs on both sides for 15 min for faster and better pore creation. Then we remove the mixture, clean the reservoirs again with DI water for at least 3 times and then add 370 μL 1 M KCl-10 mM Tris solution (Sigma-Aldrich). The KCl solution has pH ~8, and conductivity *σ*~11.5 S/m. 80 μL NaOCl solution (Sigma-Aldrich) is also added to both reservoirs. Before CT-CDB, we need to use patch clamp system (Molecular Devices) to check if the solution directly contacts the chip surface and there are no bubbles there. Usually if the intrinsic current leakage at zero external voltage is around 70 ~ 300 pA, then the device is ready to go. Otherwise, pipetting should be done to push out the bubbles until the channel is full of solution.

For CT-CDB, the device first is placed inside the double Faraday cages with a pair of Ag/AgCl electrodes inserted into two reservoirs. A DC power supply (KEYSIGHT E3631A) is turned on for external voltage with customized control circuits and a Labview program for voltage control (Figure 1b). Two main parameters need to be set carefully: initial voltage (mV) before the fabrication starts, and cut-off current (nA) during the fabrication. To make a pore with diameter of ~20 nm, it is better to start with 2500 mV for initial voltage and then keep the applied electric field to ~0.7 V/nm. Depending on the stable current level (Figure 1c, flat part of current), we usually set the cut-off current level twice or three times higher than the stable level shown on screen. Once the current surges over the cut-off level, the voltage will automatically stop. This fabrication process usually takes less than 10 min. We use episodic mode of patch clamp to record the I-V, estimate the conductance of nanopore and use equation $G\left(d_{\mathrm{pore}}\right)={\left(\frac{4L}{\sigma \pi d_{\mathrm{pore}}^{2}}+\frac{1}{\sigma d_{\mathrm{pore}}}\right)}^{-1}$ for pore size estimation [27]. If the pore size is much smaller than what we expected, 1~3 s voltage peak (initial voltage ~2000 mV) can gradually and safely enlarge the pore size to the desired one. The specific pore size should be reconfirmed as mentioned before, and the pore creation is done. ~30 min stabilization (staying still) is needed and after that, we can change the solution to pure 1 M KCl-10 mM Tris. Remeasuring the pore size, this value is the final one (Figure 1d). To further stabilize the pore, we can put the device still for extra 30~60 min depending on the noise level during the I-V measurement.

### 4.3 | Characterization of Nanopores

During CT-CDB fabrication, the initial pore size was estimated. To obtain more precise dimensional information, tapping-mode AFM (Cypher AFM) was used to measure the membrane thickness after the experiment. The results indicate that the additional 2D material layer is approximately 2–3 nm thick. Combined with the SiNx layer thickness (12 ± 2 nm), this yields an effective pore length of ~14 nm for bilayer nanopores and ~16 nm for multilayer ones (Figure S9).

Material type confirmation was conducted by SEM EDXA (JEOL JSM-IT500HR) and further by Raman Spectroscopy. Each sample was tested at room temperature on a quartz microscope slide using a Horiba Lab RAM HR confocal micro-Raman Spectrometer with a 532 nm laser set to 1% power using a 100x microscope objective lens, with Synapse Plus detector and 1800 grating. Raw data was captured and analyzed with LabSpec 6 software, and the Raman figure was created using Origin plotting software. To protect the sample from damage due to prolonged laser exposure, the spectra taken for each sample was limited to where peak signals were expected.

Scanning Transmission Electron Microscopy (STEM) of nanopore samples was also performed on a Cs corrected ThermoFisher Scientific Titan operating at 300 kV. Post-CT-CDB membranes of 11.3 μm × 11.3 μm were surveyed in TEM in a 4k × 4k Oneview CCD camera and in STEM by high-angle annular dark field detector.

### 4.4 | Transferrin Sample Preparation and Translocation Detections

Holo human serum transferrin (holo-hSTf) (Sigma–Aldrich) powder is dissolved into 1× PBS buffer. And in our experiments, we diluted the holo-hSTf solution to 100 nM for testing. Axopatch 200B-Digidata 1440A patch clamp system was utilized to detect and collect translocation signals. In general, three typical external voltages were applied: 50, 100 and 150 mV. Over 150 mV the current baseline becomes unstable with higher noise, and the device can be clogged more easily and frequently.

### 4.5 | Data Analysis Details

Single translocation events were extracted by MATLAB application EventPro 3.0 [72] in multi-level mode, which will extract more inner information of single events. The raw data has 33 features and based on them we also calculated relative current drop for further analysis. Comprehensive statistical analysis is conducted by R programs as shown in Figure 3 and Figure 4. COMSOL simulation for electric potential distribution from various nanopore structures combines static electric field and diluted species transport field, and the potential difference among those structures reveals part of the mechanism which affects the behavior of protein translocation (see Supplementary COMSOL Simulation Note).

### 4.6 | Machine Learning Based Membrane Classification

Translocation events were parameterized using engineered features including dwell time, current blockade, relative blockade, velocity, signal-to-noise ratio, and asymmetry metrics. Features were z-score normalized and used to train a stacked ensemble classifier consisting of three base learners: a multilayer perceptron (MLP), LightGBM, and extremely randomized trees and an XGBoost meta-learner. Model performance was evaluated on a stratified test set using accuracy and confusion matrices (Figure 5b; Figure S29). Feature contributions were ranked via built-in importance metrics, and model interpretability was assessed using SHAP values (Figure 5c; Figures S30 and S31). Model performance as a function of feature subset size and MLP training epochs is shown in Figures S32–S34. A detailed description of the machine learning architecture, training parameters, and evaluation procedures is provided in the Supplementary Machine Learning Note.

### 4.7 | Device Reproducibility

46 chips in total were fabricated and 36 chips sustained intact nanopore structures (no material fall-off) with stable nanopores and steady proteins translocation. Each structure includes at least 5 samples. The general yield is around 78%, which indicates the robustness of our fabrication. With the self-cleaning effect of CT-CDB, all types of nanopores can endure continuous protein tests for more than 8 h and the whole lifespan of the devices with intermittent tests can reach more than 3 days. The translocation clogging happens but can be solved by applying negative voltage or short high voltage peak (zapping). During the tests and within the device lifespan, no obvious pore size change or noise fluctuation is observed.

## Supplementary Material

supporting information

Supporting Information

Additional supporting information can be found online in the Supporting Information section.

**Supporting File**: smll73035-sup-0001-SuppMat.pdf.

## Figures and Tables

**TABLE 1 |:** Comparison of different nanopore fabrication methods and their performance.

Method	Fabrication efficiency	Structural complexity	Cost	Accuracy	In situ

TEM [30]	Low	Single Material	Very High	Single-nanometer	No
FIB [31]	Moderate	Single Material	High	~5–100 nm	No
Optical breakdown [32]	High	Single Material	Medium-high (laser+scope)	Few-nm to tens-nm; deterministic sub-5 nm with feedback	Partly
CDB [10]	High	Single Material	Low	~2–40 nm	Yes
Enhanced CT-CDB (this work)	High	Multilayer	Low	~2–30 nm	Yes

## Data Availability

The data supporting the results of this study are available in this article and its Supplementary Information. Additional raw data is available from the corresponding author upon reasonable request.
