# Supplementary material for: Heterogeneous Multilayer Nanopores via Chemically Tuned Dielectric Breakdown for Single‐Molecule Sensing
Source: Small. 2026 Mar 6;22(36):e13242. doi: 10.1002/smll.202513242 (PMC13210381; doi:10.1002/smll.202513242)
Supplement: Supplementary file 1 — Supporting File: smll73035‐sup‐0001‐SuppMat.pdf. [file SMLL-22-e13242-s001.pdf]

Supplementary Information

# Heterogeneous Multilayer Nanopores via Chemically Tuned Dielectric Breakdown for Single-Molecule Sensing

Chaoming Gu[1, †], Kamruzzaman Joty[1, †], Navod Thyashan[1], Ivan Vlassiuok[2], Liam Collins[2], Xingye Zhang[3], Christopher Nelson[2], Nathan Taton[4], Sangyoup Lee[5] & Min Jun Kim[1*]

[1]Department of Mechanical Engineering, Southern Methodist University, Dallas, Texas, USA

[2]Center for Nanophase Materials Sciences, Oak Ridge National Laboratory, Oak Ridge, Tennessee, USA

[3]College of Information Science and Electronic Engineering, Zhejiang University, Hangzhou, Zhejiang, China

[4]Department of Chemistry, Southern Methodist University, Dallas, Texas, USA

[5]Bionic Research Center, Biomedical Research Division, Korea Institute of Science and Technology, Seoul, Republic of Korea

[†]These authors contributed equally

*Correspondence Author mjkim@lyle.smu.edu

**Table of Contents**


Supplementary Tables ........ 1

Table S 1 Parameters of different materials in simulations. ........ 1

Table S 2 Engineered features utilized for machine learning classification........ 2

Table S 3 Translocation event count and train-test distribution by membrane type. ........ 3

Supplementary Figures ........ 4

Figure S 1 Cross-sectional schematics of multilayer devices ........ 4

Figure S 2 Representative clogging traces from ~16 nm pores at 50 mV. ........ 5

Figure S 3 Comparison of fabrication time among different fabrication methods. ........ 6

Figure S 4 Scanning electronic microscope energy dispersive X-ray analysis for bilayer composition characterization. ........ 7

Figure S 5 Scanning electronic microscope energy dispersive X-ray analysis for tri-layer composition characterization. ........ 8

Figure S 6 Raman spectroscopy of SH, SG, SM, SGH, and SGM structures represented by purple, green, blue, red and black spectra, respectively. ........ 9

Figure S 7 Scanning transmission electron microscopy analysis of multilayer nanopore membrane surfaces post CT-CDB. ........ 10

Figure S 8 Atomic force microscopy (AFM) analysis of SH and SM membrane surfaces following CT-CDB nanopore fabrication. ........ 11

Figure S 9 The thickness measurements of $MoS_2$ and graphene by AFM. ........ 12

Figure S 10 Comparison of normalized Hooge parameter across structures, voltages, and devices. ..... 13

Figure S 11 Comparison of 1/f noise level from different nanopore fabrication methods. ........ 15

Figure S 12 Representative ionic current traces from bilayer and $SiN_x$ nanopores at 50 mV and 150 mV bias. ........ 16

Figure S 13 The scattered distribution between relative current drop and dwell time across all bilayer devices. ........ 18

Figure S 14 Capture rate of different bilayer structures across all devices and voltages. ........ 19

Figure S 15 The scattered distribution between relative current drop and dwell time across all tri-layer devices. ........ 20

Figure S 16 Comparative analysis of relative current drop between SGH and SH across all devices and voltages. ........ 21

Figure S 17 Comparative analysis of dwell time between SGH and SGM across all devices and voltages. ........ 22

Figure S 18 Comparative analysis of dwell time between SGH and SH across all devices and voltages. 23

Figure S 19 Comparative analysis of relative current drop between SGM and SM across all devices and voltages. ........ 24
Figure S 20 Comparative analysis of dwell time between SGM and SM across all devices and voltages. ........ 25
Figure S 21 Capture rate comparison among tri-layer nanopores and bare $SiN_x$ nanopores. ........ 26
Figure S 22 COMSOL simulation for electric potential difference among various nanopore structures at 100 mV. ........ 27
Figure S 23 Diagram of the mechanism governing biomolecule translocation in multilayer nanopores. . 28
Figure S 24 Annotated sample ionic current trace with extracted features. ........ 29
Figure S 25 Accuracy comparison of individual classifiers. ........ 30
Figure S 26 Evaluation of stacked model variants using different base learner combinations. ........ 31
Figure S 27 Accuracy ranking of the top 12 (excluding final model) stacked meta-model configurations. ........ 32
Figure S 28 Confusion matrix of the stacked model evaluated on a unique $SiN_x$-only dataset. ........ 33
Figure S 29 Confusion matrix showing absolute event counts classified by the ensemble model. ........ 34
........ 35
Figure S 30 Importance scores of all 47 features utilized for classification. ........ 35
Figure S 31 SHAP (SHapley Additive exPlanations) summary plot for model interpretability. ........ 36
Figure S 32 Classification accuracy of XGBoost versus number of top-ranked features used. ........ 37
Figure S 33Classification accuracy of LightGBM as a function of the number of top-ranked features. ... 38
Figure S 34 MLP classifier accuracy versus number of training epochs. ........ 39
Supplementary Notes ........ 40
Volume Exclusion Model Note ........ 40
COMSOL Simulation Note ........ 41
Machine Learning Note ........ 43
Reference ........ 44

# Supplementary Tables

**Table S 1 Parameters of different materials in simulations.**

This table lists key material property parameters, including relative permittivity[1,2], electric conductivity, and surface charge[3–6].

| Material | Relative permittivity | Electrical conductivity S/m | Surface charge C/m$^2$ |
|---|---|---|---|
| **$SiN_x$** | 7 | 0 | $-2\times10^{-2}$ |
| **hBN** | 3.44 | 0 | $-5\times10^{-4}$ |
| **$MoS_2$** | 6.9 | $3\times10^{-3}$ | $-5\times10^{-2}$ |
| **Graphene** | 3 | $10^{6}$ | $-3\times10^{-2}$ |

**Table S 2 Engineered features utilized for machine learning classification.**

This table lists and defines the full set of 47 engineered features derived from individual protein translocation events. Features span across three domains: signal shape (e.g., dwell time, current blockade, skewness), signal quality (e.g., signal-to-noise ratio, roughness), and inferred biophysical dynamics (e.g., velocity, ECD rate). These features were systematically extracted using automated routines and used to train and evaluate the classification model. Equations for each feature are included for reproducibility.

| Feature | Equation | |
|---|---|---|
| **Relative Blockade** | Current Blockade (pA) / Event Baseline (pA) | |
| **Fitted Relative Blockade** | Fitted Current Blockade (pA) / Event Baseline (pA) | |
| **Dwell Time Std** | $\sigma_{dt}=\sqrt{\frac{1}{N}\sum_{i=1}^{N}(x_i-\bar{x})^2}$ | $x_i$: Individual values in Dwell Time steps<br>$\bar{x}$: Mean of Dwell Time steps<br>N: Number of steps |
| **Dwell Time Skewness** | $S_{dt}=\frac{\frac{1}{N}\sum_{i=1}^{N}(x_i-\bar{x})^3}{\left(\frac{1}{N}\sum_{i=1}^{N}(x_i-\bar{x})^2\right)^{3/2}}$ | |
| **Dwell Time Kurtosis** | $K_{dt}=\frac{\frac{1}{N}\sum_{i=1}^{N}(x_i-\bar{x})^4}{\left(\frac{1}{N}\sum_{i=1}^{N}(x_i-\bar{x})^2\right)^{2}}-3$ | $x_i$: Individual values Current Blockade steps<br>$\bar{x}$: Mean of Current Blockade steps<br>N: Number of steps |
| **Blockade Variance** | $\sigma_{dI}{}^2=\frac{1}{N}\sum_{i=1}^{N}(x_i-\bar{x})^2$ | |
| **Blockade Depth Threshold** | $dI_{th,i}=0.5\times dI_{fit,i}$ | $dI_{fit,i}$: Fitted blockade depth for event $i$ |
| **Multi-Level Index** | $\frac{\sum_{j=1}^{N}1\left(dI_{j,i}>dI_{th,i}\right)}{\text{Level Count}_i}$ | $dI_{j,i}$: Blockade depth at level $j$ for event $i$<br>$1\left(dI_{j,i}>dI_{th,i}\right)$: Indicator function (1 if condition is met, 0 otherwise)<br>N: Total number of levels |
| **Deepest Blockade Level** | Max of Current Blockade Columns | |
| **Blockade Asymmetry** | $\frac{dI_{1,i}-dI_{N,i}}{dI_i}$ | $dI_{1,i}$: Initial blockade depth<br>$dI_{N,i}$: Final blockade depth |
| **Fitted Blockade Asymmetry** | $\frac{dI_{1,i}-dI_{N,i}}{dI_{fit,i}}$ | $dI_{fit,i}$: Fitted blockade depth |
| **Signal to Noise** | Fitted Current Blockade (pA) / Windowed Stdv. (pA) | |
| **Dwell Asymmetry** | $\frac{dt_{1,i}-dt_{N,i}}{dt_i}$ | $dt_{1,i}$: Initial dwell time<br>$dt_{N,i}$: Final dwell time |
| **Fitted Dwell Asymmetry** | $\frac{dt_{1,i}-dt_{N,i}}{dt_{fit,i}}$ | $dt_{fit,i}$: Fitted dwell time |
| **Fitted ECD Rate** | ECD (fC) / FWHM Dwell Time(ms) | |
| **Blockade Slope** | $\frac{\sum_{j=1}^{N}(x_j-\bar{x})\left(dI_{j,i}-\bar{dI}\right)}{\sum_{j=1}^{N}(x_j-\bar{x})^2}$ | $x_j$: Sequential index (1 to $N$)<br>$\bar{x}$: Mean of $x_j$<br>$\bar{dI}$: Mean blockade depth |
| **Blockade Roughness** | $\frac{\sigma_{dI,i}}{dI_i}$ | |
| **Velocity (nm/ms)** | Membrane thickness (nm) / E2E Dwell Time (ms) | |

**Table S 3 Translocation event count and train-test distribution by membrane type.**

This table reports the total number of protein translocation events extracted from each of the six membrane types: bare $SiN_x$, $SiN_x$+Graphene (SG), $SiN_x$+$MoS_2$ (SM), $SiN_x$+hBN (SH), $SiN_x$+Graphene+$MoS_2$ (SGM), and $SiN_x$+Graphene+hBN (SGH). Events were randomly divided into training (80%) and testing (20%) sets[7], ensuring balanced class representation and minimizing bias in classification performance evaluation. Our data sets have more than 35k events, and the total amount is enough to the machine learning requirement.

| **Event distribution** | **Number of events** | | |
|---|---|---|---|
| **Membrane type** | **Full dataset** | **Train set** | **Test set** |
| **SGH** | 87711 | 65783 | 21928 |
| **SG** | 58473 | 46778 | 11695 |
| **SM** | 51876 | 38907 | 12969 |
| **$SiN_x$** | 41226 | 30919 | 10307 |
| **SGM** | 39551 | 29663 | 9888 |
| **SH** | 36338 | 27254 | 9084 |

## Supplementary Figures

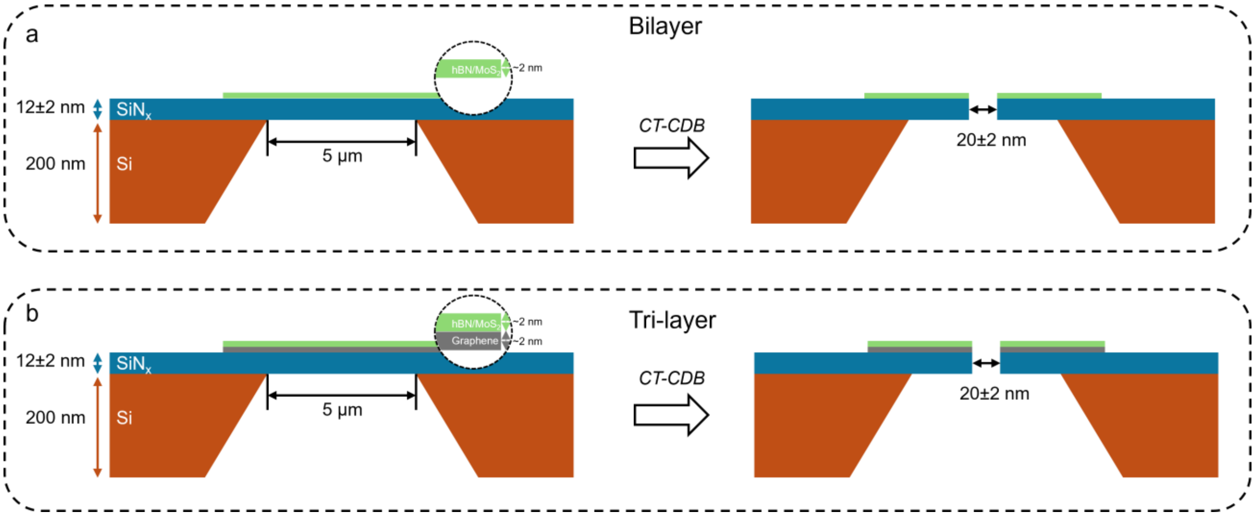


**Figure S 1 Cross-sectional schematics of multilayer devices**

(a) and (b) illustrate the schematics of the bilayer and tri-layer structures, respectively. The critical device geometric parameters are included and labeled.

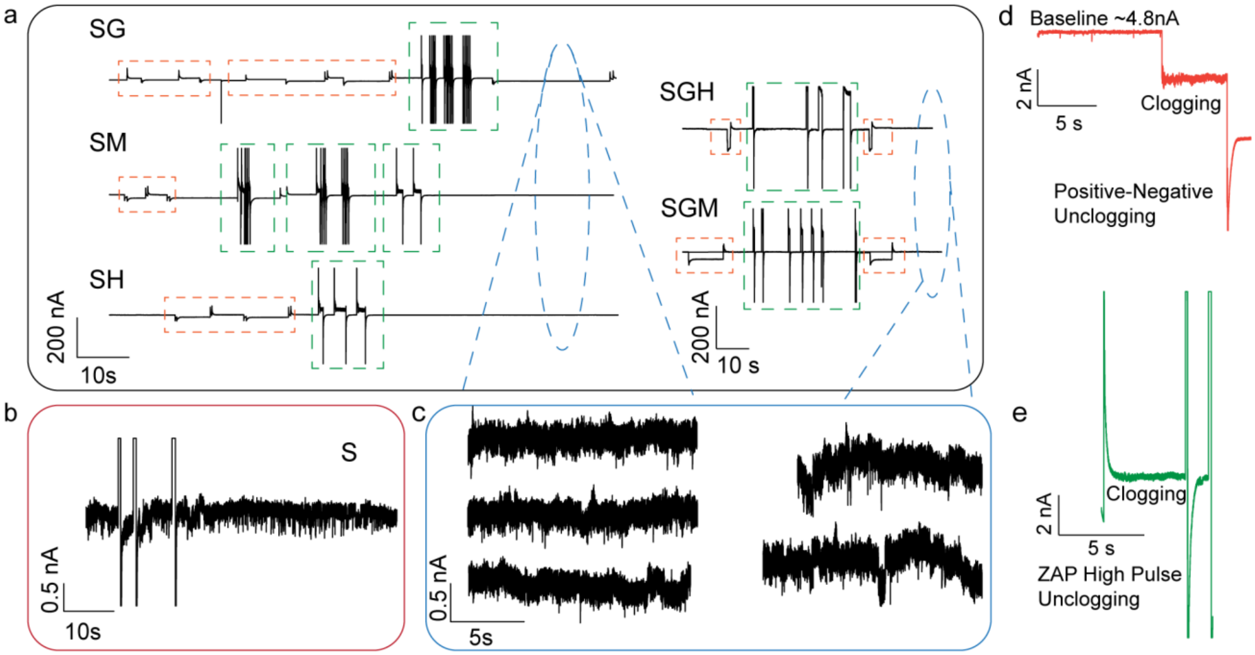


**Figure S 2 Representative clogging traces from ~16 nm pores at 50 mV.**

(a) Shows clogging traces from 5 multilayer nanopore structures with ~16 nm pore diameter. Red and green rectangles mark the clogging resolution processes using (d) positive-Negative unclogging and (e) ZAP high pulse unclogging, respectively. The frequent appearance of up and down peaks indicates that the normal baseline cannot be recovered due to transferrin clogging. (b) Shows a representative clogging trace from SiN nanopore. (c) Exhibit trace segments after clogging. The baseline stability is severely compromised, and no translocation events can be observed.

This frequent clogging issue severely reduces the number of analyzable translocation events and prevents statistically meaningful ensemble analysis. As a result, nanopores with nominal effective diameters below ~18 nm were excluded from the main dataset, and pores with diameters of ~20 ± 2 nm were selected as the operational regime. This range balances stable protein transport, sufficient capture rates, and device robustness for multilayer architectures.

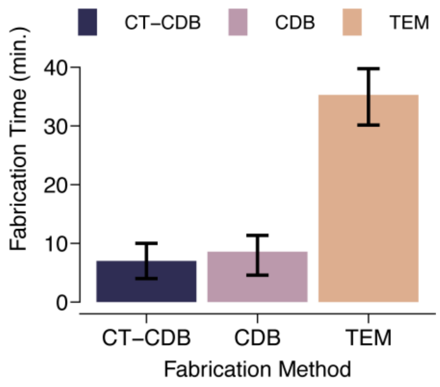


**Figure S 3 Comparison of fabrication time among different fabrication methods.**

The fabrication time of CT-CDB (this work), CDB and TEM to create a ~20 nm pore. Among three fabrication methods, CT-CDB can create a multilayer pore within 10 minutes. Different multilayer structures need similar time to drill a pore. For CDB, the fabrication time is similar to CT-CDB, but we can only get a ~20 nm pore with bad quality (baseline stability, noise level etc.). And for TEM, including gas-pumping, window finding and focusing, at least 30 minutes are needed to drill a pore.

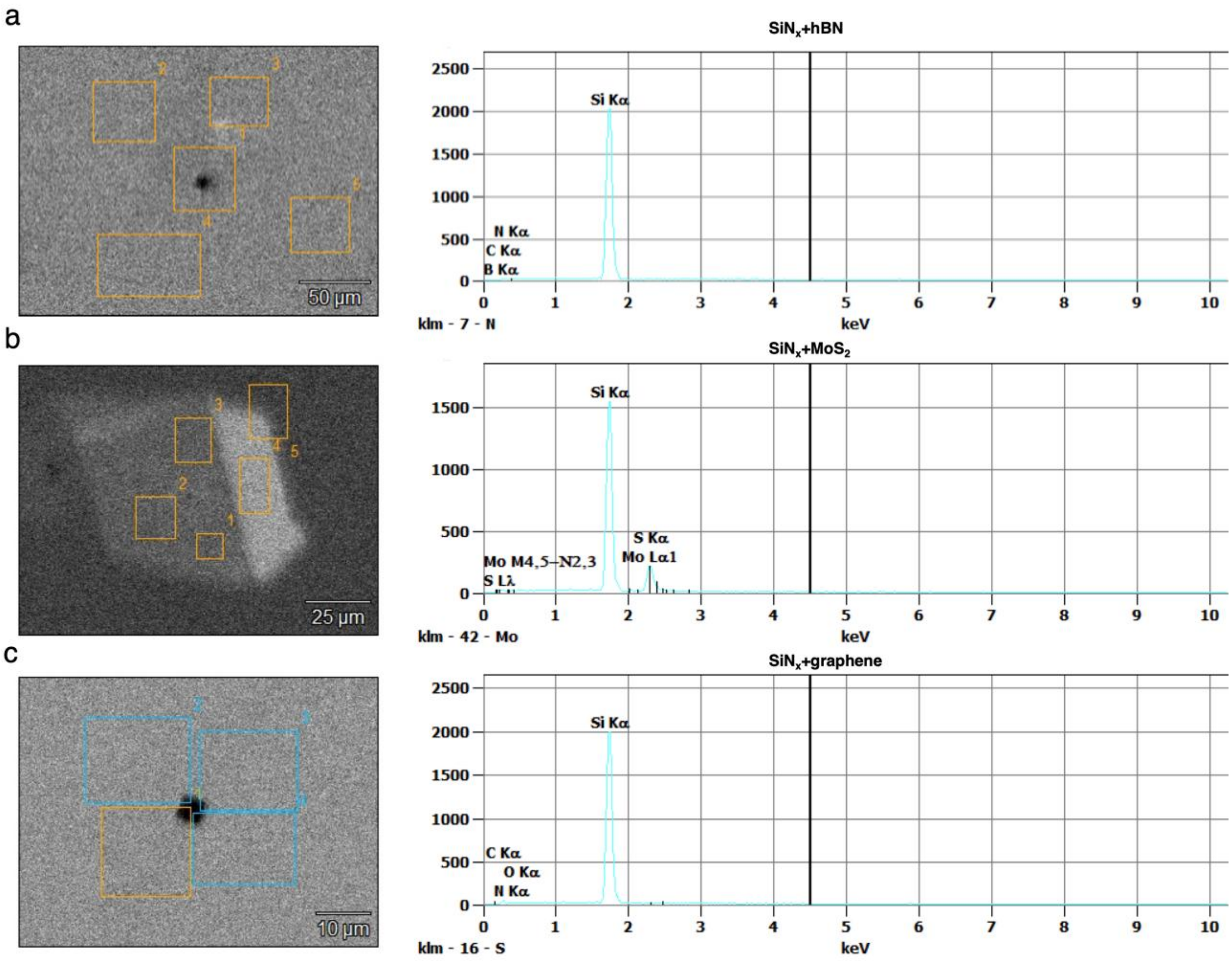


**Figure S 4 Scanning electronic microscope energy dispersive X-ray analysis for bilayer composition characterization.**

(a), (b) and (c) show the SEM observations on the left, and the corresponding composition analysis results on the right, with the bilayer structure SH, SM, and SG respectively. The energy of the Silicon is the highest since it is the thickest part. Due to the 2D property, extra layer on top of $SiN_x$ has weak energy signals. Nevertheless, the appearance of energy of all corresponding elements verifies the intactness of our bilayer structures with correct materials. The black square area is the free-standing window area where the nanopore lies through.

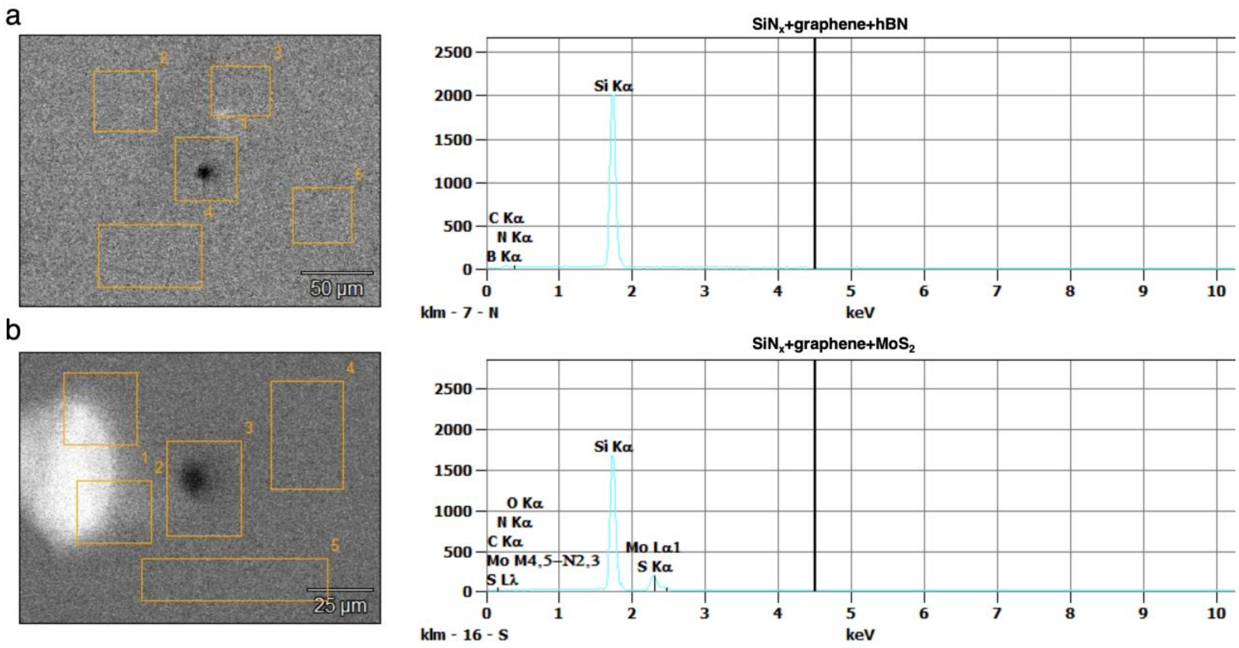


**Figure S 5 Scanning electronic microscope energy dispersive X-ray analysis for tri-layer composition characterization.**

(a) and (b) shows the SEM EDXA results of SGH and SGM tri-layer structure. The analysis results exhibit both the graphene middle layer (carbon) and the top layer element compositions. Similar to bilayer structure, the energy of other elements is relatively weak except silicon, but the results can still prove the tri-layer structures are intact.

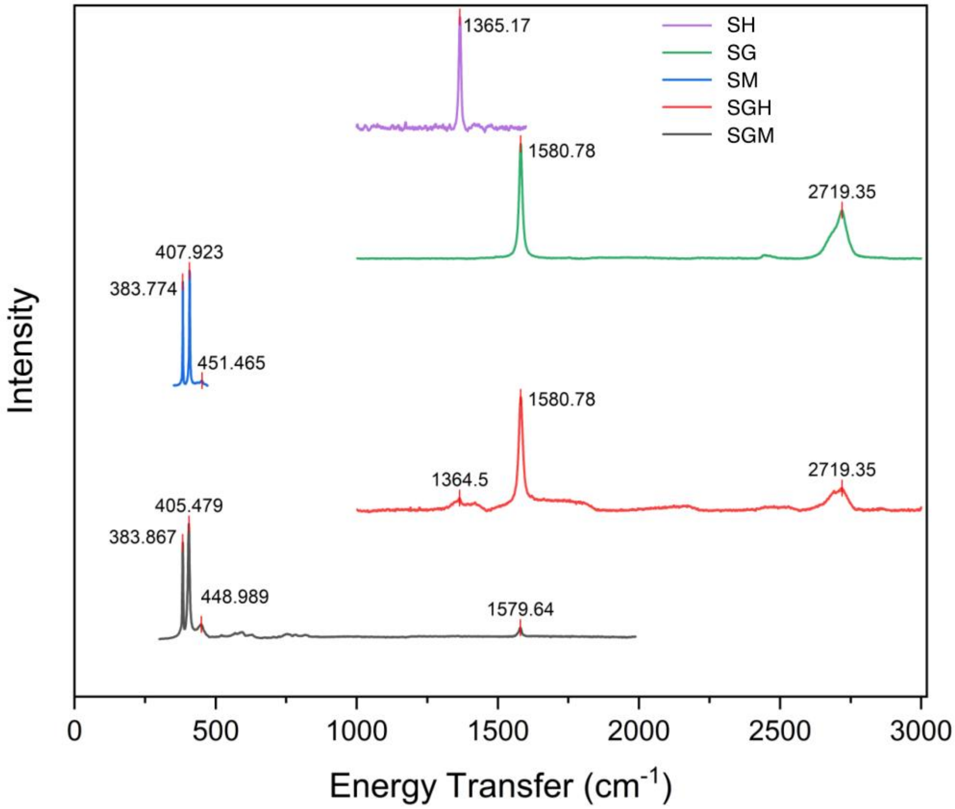


**Figure S 6 Raman spectroscopy of SH, SG, SM, SGH, and SGM structures represented by purple, green, blue, red and black spectra, respectively.**

The Raman Spectroscopy is conducted for all multilayer structures to verify the materials at 532 nm laser. SH exhibits one peak at 1365.75 cm$^{-1}$, showing the typical 2D hBN characteristic peak[8] with 2~3 layers. SG shows two peaks at 1580.78 and 2719.35 cm$^{-1}$. The first peak is the G peak and the second is the 2D peak[9], indicating the pristine state of the graphene. There are two $MoS_2$ typical $E^1_{2g}$ (383.774 cm$^{-1}$) and $A_{1g}$ (407.923 cm$^{-1}$) peaks appeared from SM structure[10], and the extra peak at 451.465 cm$^{-1}$ is the 2LA band from second-order phonon process[11]. SGH shows three peaks. First peak at 1364.5 cm$^{-1}$ confirms the appearance of hBN and the last two peaks, same as SG structure, correspond to the graphene layer. For SGM one, the first three peaks (383.867, 405.479 and 448.989 cm$^{-1}$) indicate the existence of $MoS_2$ layer. But in this case, the graphene layer only shows the G peak at 1579.64 cm$^{-1}$ without the 2D peak. Since $MoS_2$ absorbed most of the laser intensity, 2D peak of graphene underneath is decreased[12]. The spectra intensities have been normalized and offset for clarity.

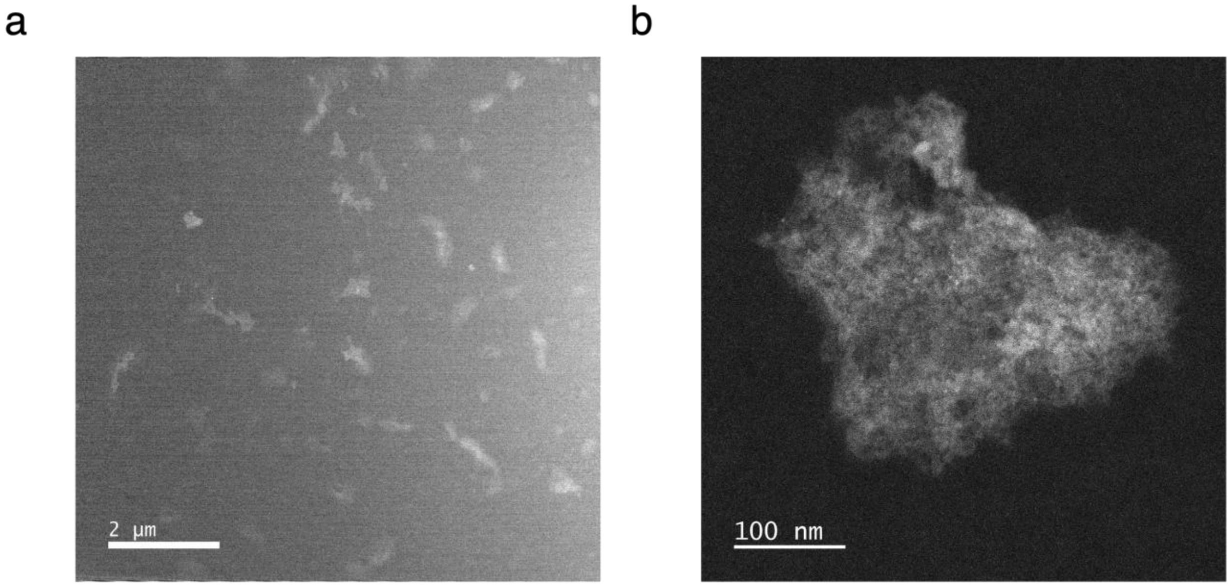


**Figure S 7 Scanning transmission electron microscopy analysis of multilayer nanopore membrane surfaces post CT-CDB.**

STEM micrographs of multilayer membrane regions. (a) Lower magnification TEM shows the free-standing membrane along with prominent surface-adhered polymeric structures (white features). (b) A higher magnification view of one such feature via high-angle annular dark field (HAADF)-STEM. The entire membrane was scanned at 2nm/pixel resolution using this HAADF-STEM mode. It is sensitive to the mass-thickness, a region of vacuum (a pore) would appear as a dark region. However, a projected vacuum region was not found, presumably the significant coverage by surface contaminants has occluded the pore region. The presence of these residues highlights a common challenge in post-measurement chip recovery and limits direct TEM-based identification of nanopore openings in multilayer configurations.

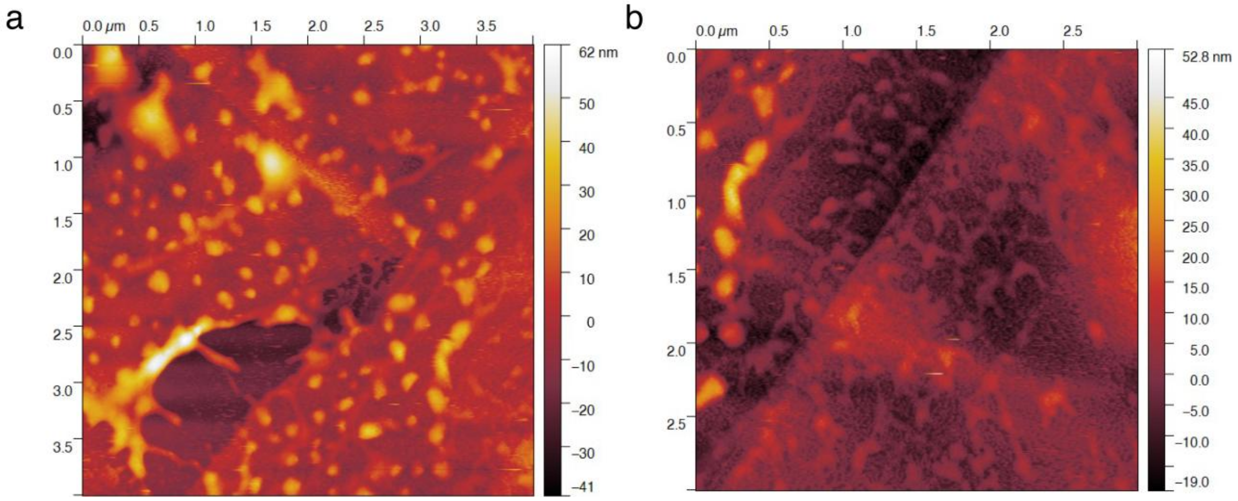


**Figure S 8 Atomic force microscopy (AFM) analysis of SH and SM membrane surfaces following CT-CDB nanopore fabrication.**

(a) Surface topography of a $SiN_x$+hBN (SH) membrane. (b) Surface topography of a $SiN_x$+$MoS_2$ (SM) membrane.

Both membranes exhibit widespread nanoscale surface features consistent with adsorbed contaminants or phase-change artifacts. These structures, possibly polymeric or salt-based in origin, are difficult to remove after device retrieval and may arise from drying-induced deposition or fluid–surface interactions during post-experiment handling. While such surface residues can hinder high-resolution imaging and AFM-based pore identification, they do not interfere with single-molecule translocation measurements conducted in the liquid phase, provided the membrane architecture remains stable and fully immersed.

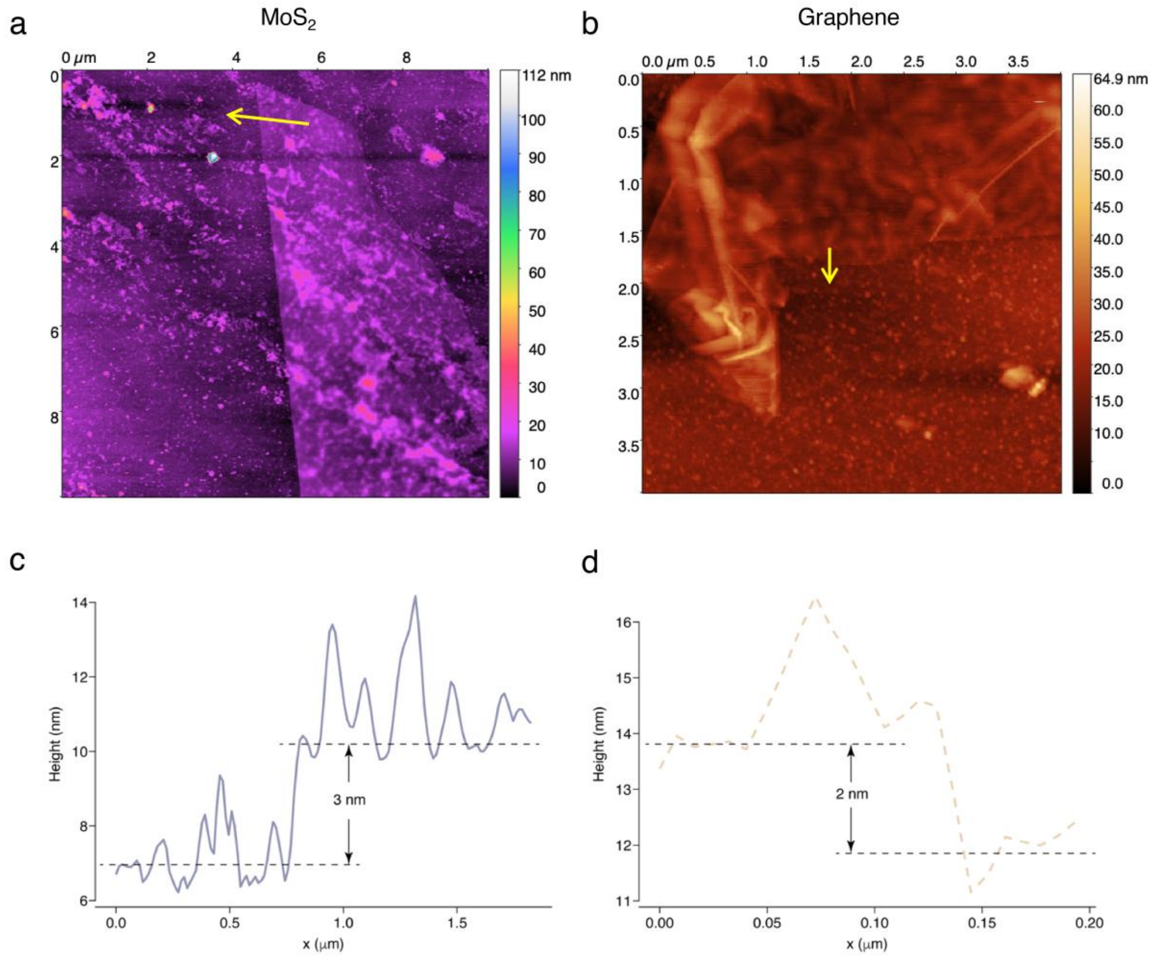


**Figure S 9 The thickness measurements of $MoS_2$ and graphene by AFM.**

(a) AFM height map of an exfoliated $MoS_2$ flake on a $SiN_x$ substrate. The yellow arrow indicates the position and direction of the thickness line scan. (b) AFM image of an exfoliated graphene layer with the corresponding line scan path indicated. (c) Height profile along the arrow in panel a, showing a step height of approximately 3 nm, consistent with multilayer $MoS_2$. (d) Height profile along the arrow in sub-figure **b** yielding a thickness of approximately 2 nm, consistent with a few-layer graphene sheet.

Contamination patterns similar to those seen in Figure S6 are present on both surfaces, likely introduced during post-transfer handling or drying. For accurate thickness measurement, regions with minimal surface roughness and reduced debris were selected. Localized peaks in the profiles correspond to residual contaminants or adsorbed material, while the baseline step heights reflect the true thickness of the transferred 2D layers.

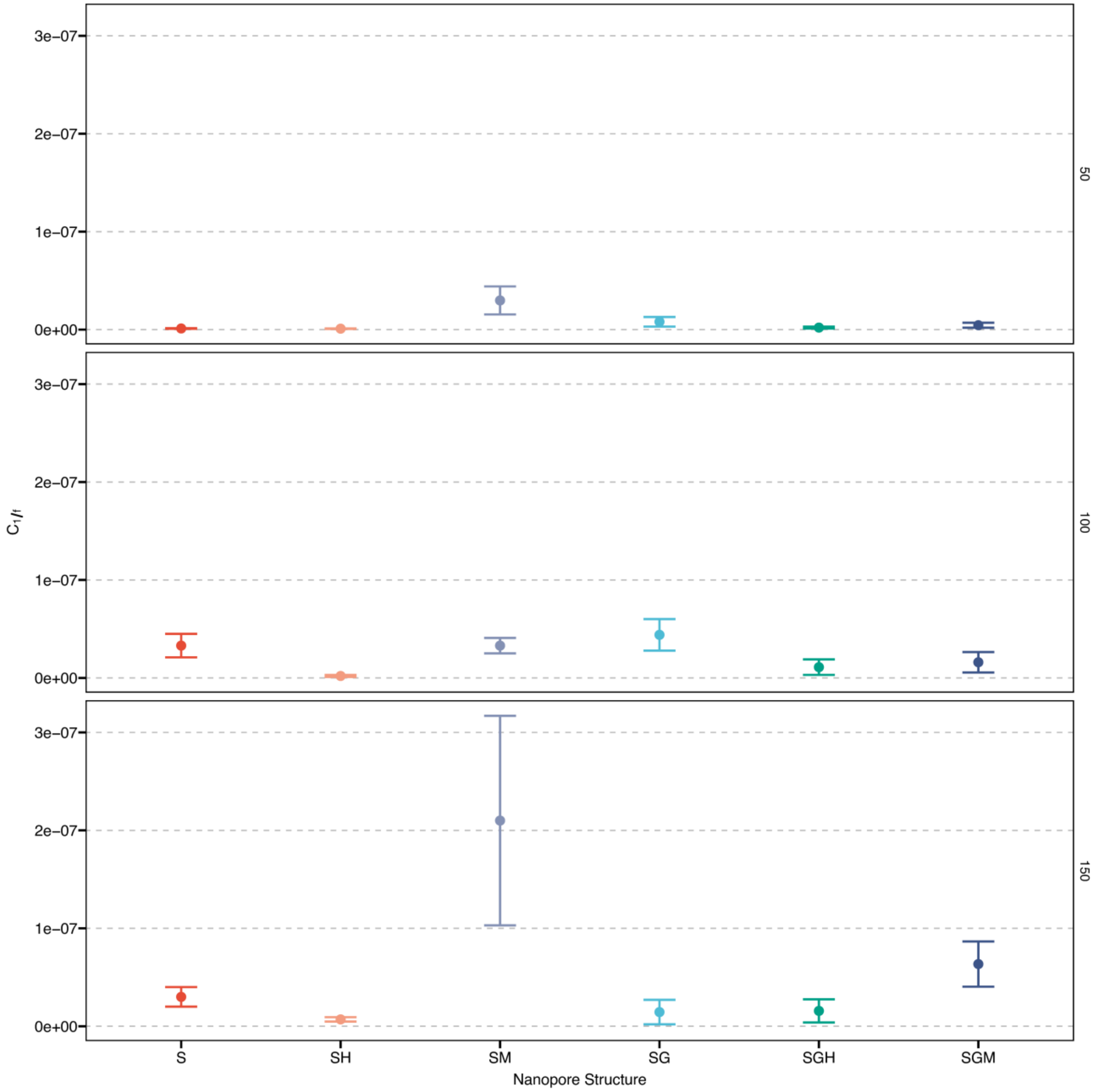


**Figure S 10 Comparison of normalized Hooge parameter across structures, voltages, and devices.**

The normalized Hooge parameter ($C_H/I^2$) is evaluated for different multilayer nanopore architectures (S, SH, SM, SG, SGH, and SGM) under applied voltages of 50, 100, and 150 mV. The Hooge parameter is a common metric to quantify 1/f noise in nanopores, and its normalization by the square of the current removes bias from conductance variations, allowing intrinsic comparison of noise sources across structures.

At 50 mV, most structures exhibit low values close to zero, except for SM, which shows a moderate increase. SH remains at a very low level, indicating that the presence of hBN effectively suppresses low-frequency noise even at low bias.

At 100 mV, the difference becomes clearer: while SM and SG show elevated noise, SH and SGH maintain suppressed Hooge values. This strongly suggests that hBN plays a stabilizing role, likely due to its atomically flat surface and chemically inert nature, which reduce charge trapping and interfacial fluctuations. Graphene-containing structures without hBN (SG) show intermediate noise, while the incorporation of hBN with graphene (SGH) again suppresses the noise back to levels comparable to pure SiN.

At 150 mV, the contrast between structures is most pronounced. SM displays a very large increase with significant variability, reflecting enhanced trap-assisted fluctuations in $MoS_2$. In contrast, both SH and SGH remain among the lowest-noise structures across all voltages. Importantly, even when graphene is present (SG vs. SGH), the addition of hBN markedly reduces noise. This demonstrates that the noise suppression is not solely due to graphene's conductivity but is strongly dependent on the inclusion of hBN as a dielectric stabilizer.

These results indicate that hBN consistently suppresses 1/f noise in solid-state nanopores, both in bilayer hybrids (SH) and in more complex stacks (SGH). While $MoS_2$ introduces significant noise, and graphene provides partial stabilization, the most effective suppression is achieved in architectures that incorporate hBN, highlighting its critical role in designing low-noise multilayer nanopores for single-molecule detection.

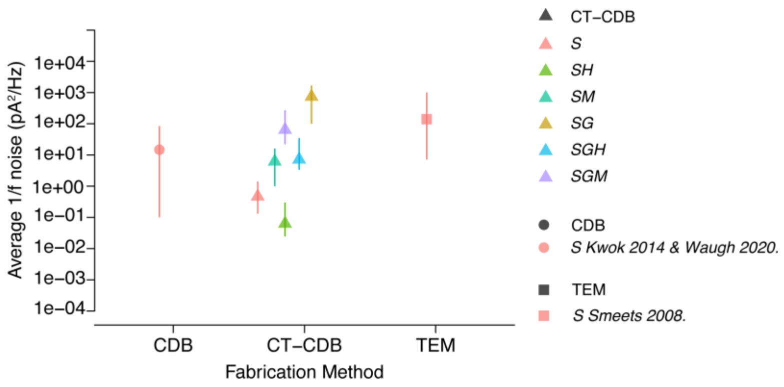


**Figure S 11 Comparison of 1/f noise level from different nanopore fabrication methods.**

Average 1/f comparison among three methods with different structures. Using $SiN_x$ nanopores as the benchmark, pore made by CT-CDB has the lowest 1/f noise (close to $10^{-1}$ $pA^2/Hz$). SH structure further suppresses the noise, but other structures increase the noise to different extent. However, the noise-changing trend should be similar. Therefore, if bare $SiN_x$ nanopores already have higher 1/f noise, the rest of the structures should also have higher noise level accordingly.

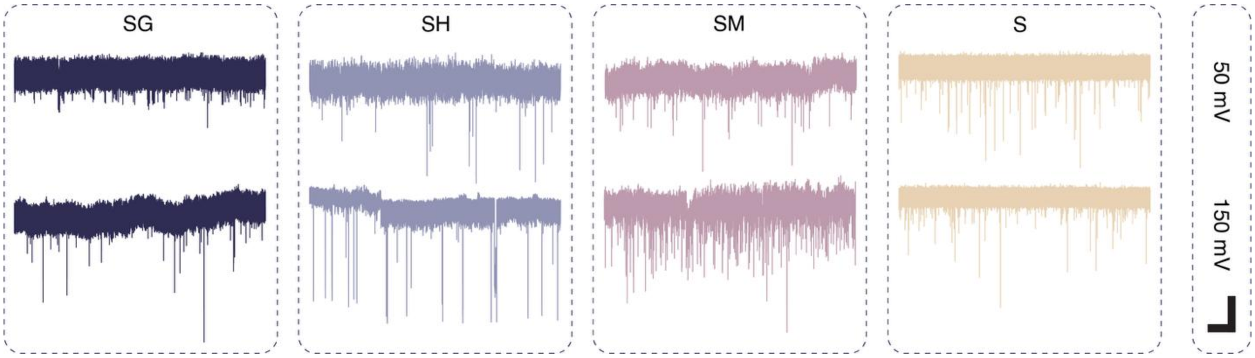


**Figure S 12 Representative ionic current traces from bilayer and $SiN_x$ nanopores at 50 mV and 150 mV bias.**

Current traces recorded over 10 seconds from nanopores composed of $SiN_x$+graphene (SG), $SiN_x$+hBN (SH), $SiN_x$+$MoS_2$ (SM), and bare $SiN_x$ (S) at 50 mV (top row) and 150 mV (bottom row). Scale bars: horizontal, 1 s; vertical, 100 pA.

Compared to other membranes, SG nanopores display the lowest capture rate and a relatively poor signal-to-noise ratio (SNR), consistent with results shown in Figure 3c. SM nanopores exhibit elevated baseline noise, particularly at higher voltage, whereas SH and bare $SiN_x$ nanopores yield clean, high-SNR translocation events with sharp and well-defined signatures. These differences highlight the influence of membrane composition on electrical noise characteristics and translocation signatures.

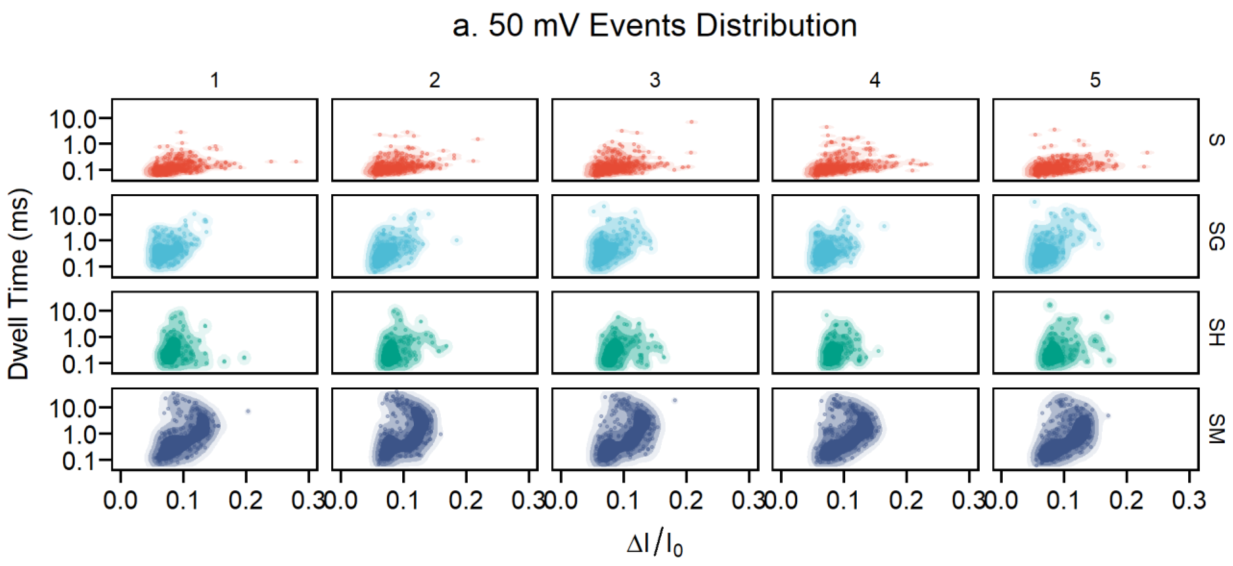
a. 50 mV Events Distribution
1
2
3
4
5
S
SG
SH
SM
Dwell Time (ms)
10.0
1.0
0.1
0.0
0.1
0.2
0.3
ΔI/I0

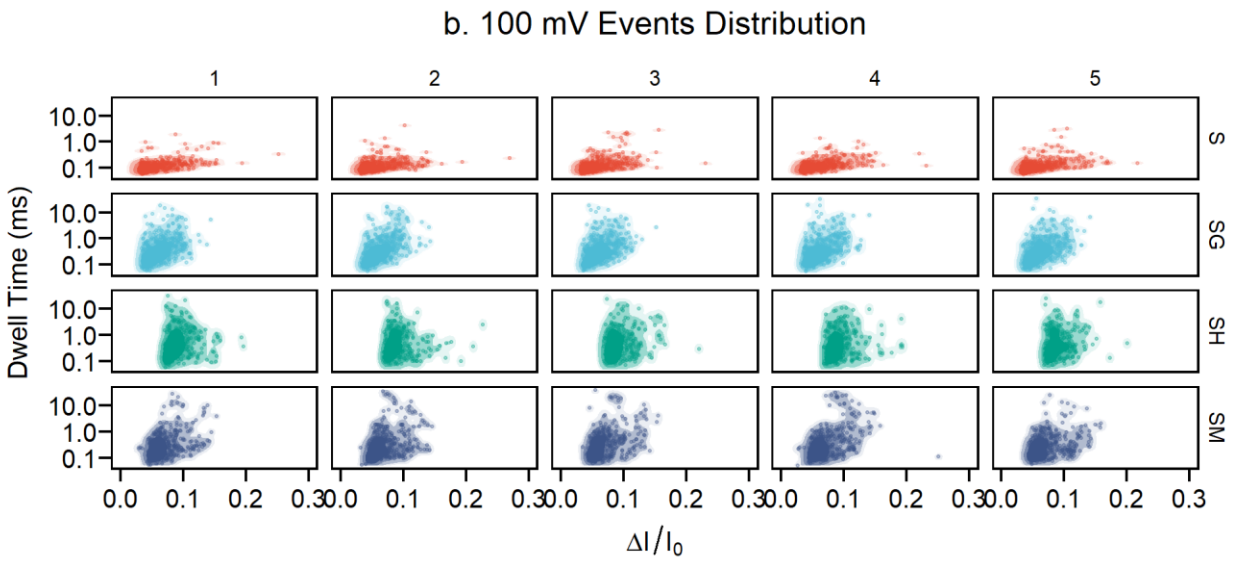
b. 100 mV Events Distribution
1
2
3
4
5
S
SG
SH
SM
Dwell Time (ms)
10.0
1.0
0.1
0.0
0.1
0.2
0.3
ΔI/I0

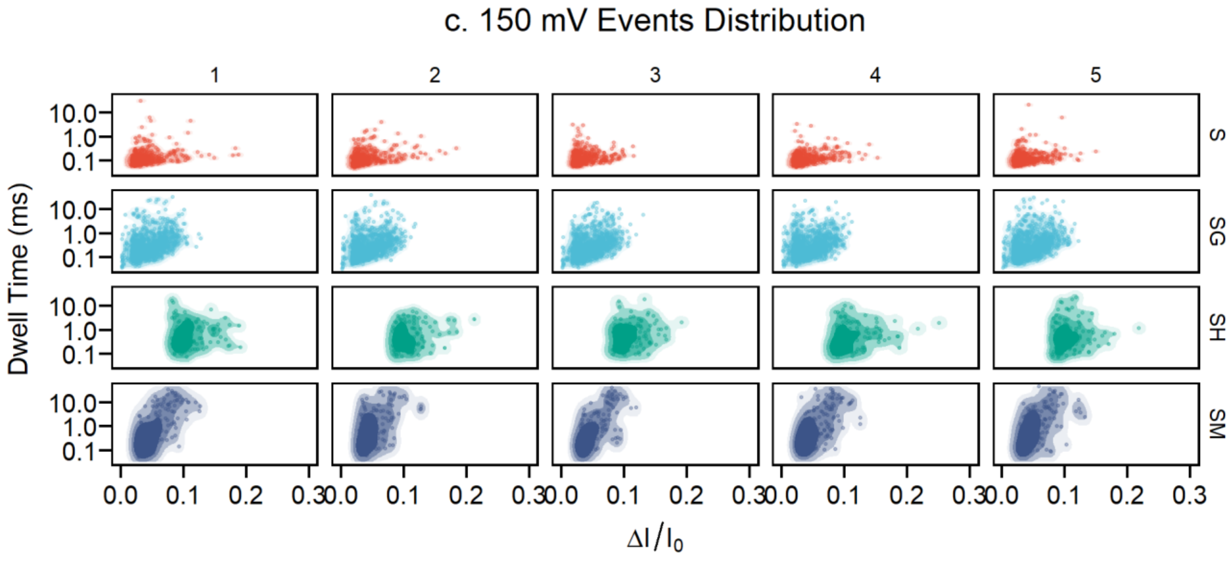


**Figure S 13 The scattered distribution between relative current drop and dwell time across all bilayer devices.**

(a) Events distribution at 50 mV. (b) Events distribution at 100 mV. (c) Events distribution at 150 mV.

Each point represents a single translocation event plotted as relative current blockade ($\Delta I/I_0$) versus dwell time on logarithmic axes. At 50 mV, the clusters corresponding to different membrane types overlap significantly, resulting in blurred boundaries and reduced class separability. In contrast, at 150 mV, distinct clustering emerges, indicating enhanced membrane-specific translocation behavior at higher driving voltage. Notably, events from SG ($SiN_x$+graphene) nanopores remain broadly distributed across both conditions, while other membrane types exhibit more compact and separable distributions, highlighting the influence of membrane composition on translocation dynamics. At one certain external voltage, the distributions are consistent across different devices for one type of structure. This consistency validates the robustness of CT-CDB on bilayer nanopore fabrication.

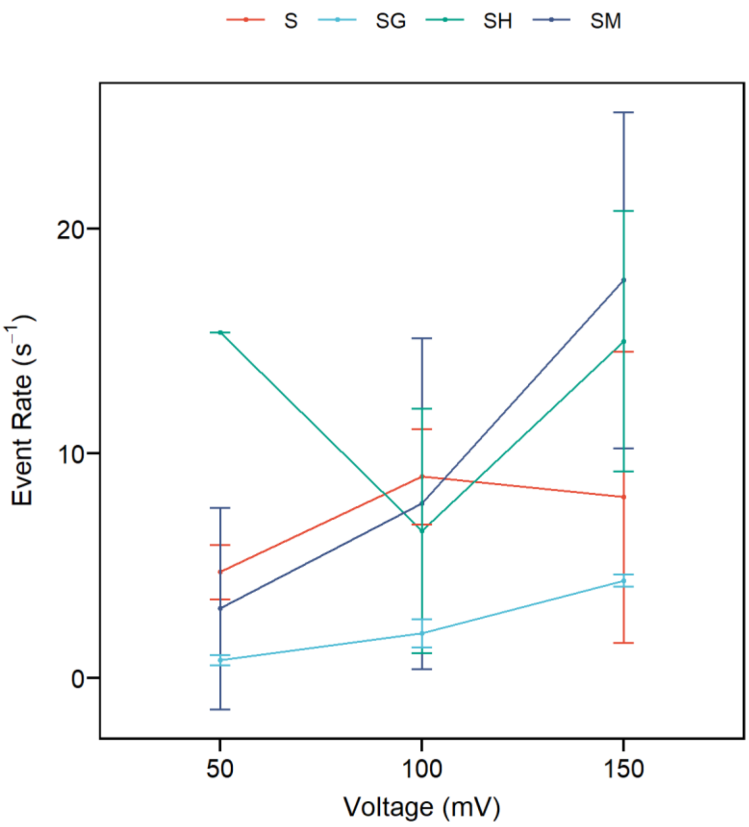


**Figure S 14 Capture rate of different bilayer structures across all devices and voltages.**

Events capture rate from bilayer and $SiN_x$ nanopores at 50, 100 and 150 mV. Among them, SG has lowest capture rate (light blue) while the other three types have similar and reasonable capture rate.

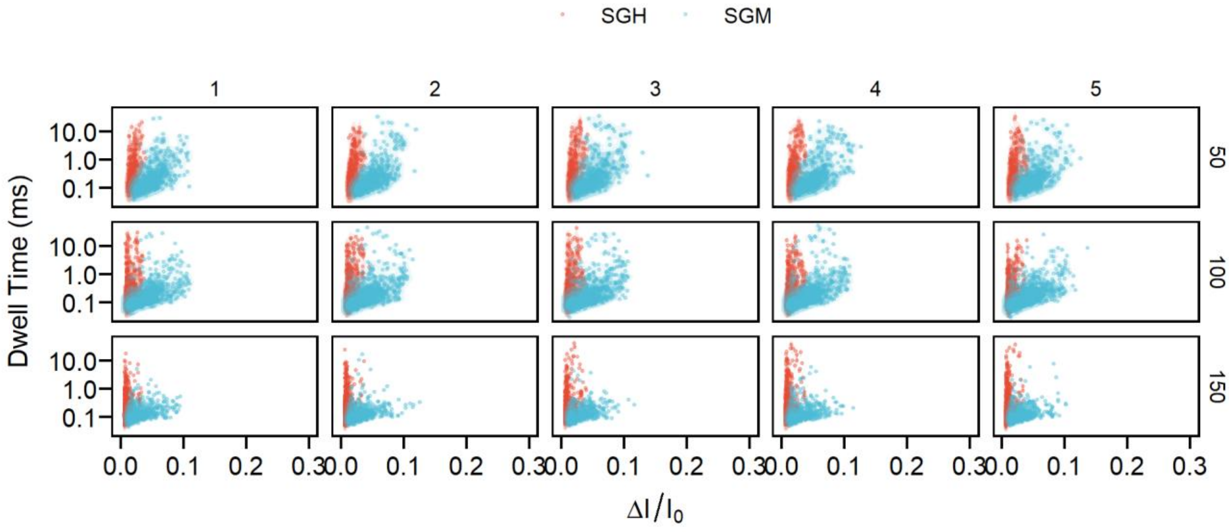


**Figure S 15 The scattered distribution between relative current drop and dwell time across all tri-layer devices.**

Translocation events through SGH ($SiN_x$–graphene–hBN, red) and SGM ($SiN_x$–graphene–$MoS_2$, blue) nanopores are compared at applied voltages of 50, 100, and 150 mV across five replicate devices. Each scatter plot shows the relationship between relative current blockade ($\Delta I/I_0$) and dwell time (log scale). Both structures exhibit a broad distribution of events, but with systematic differences:

The dwell time of SGM events tend to cluster at shorter dwell times compared to SGH, which consistently shows longer and more dispersed translocations. Both SGH and SGM reach similar relative current blockade depths (up to ~0.3), though SGM events more frequently extend into higher blockade values. Increasing the bias from 50 to 150 mV shifts the distribution of SGM toward smaller $\Delta I/I_0$ and shorter dwel, time, while events from SGH are relatively stable. Across five replicates, SGH consistently produces narrower and less scattered distributions compared to SGM, highlighting greater stability and lower variability.

These observations suggest that hBN-containing structures (SGH) promote more uniform and stable protein translocation signals, whereas $MoS_2$-containing pores (SGM) generate longer, more variable events, consistent with additional interactions or charge-trap effects in $MoS_2$ layers.

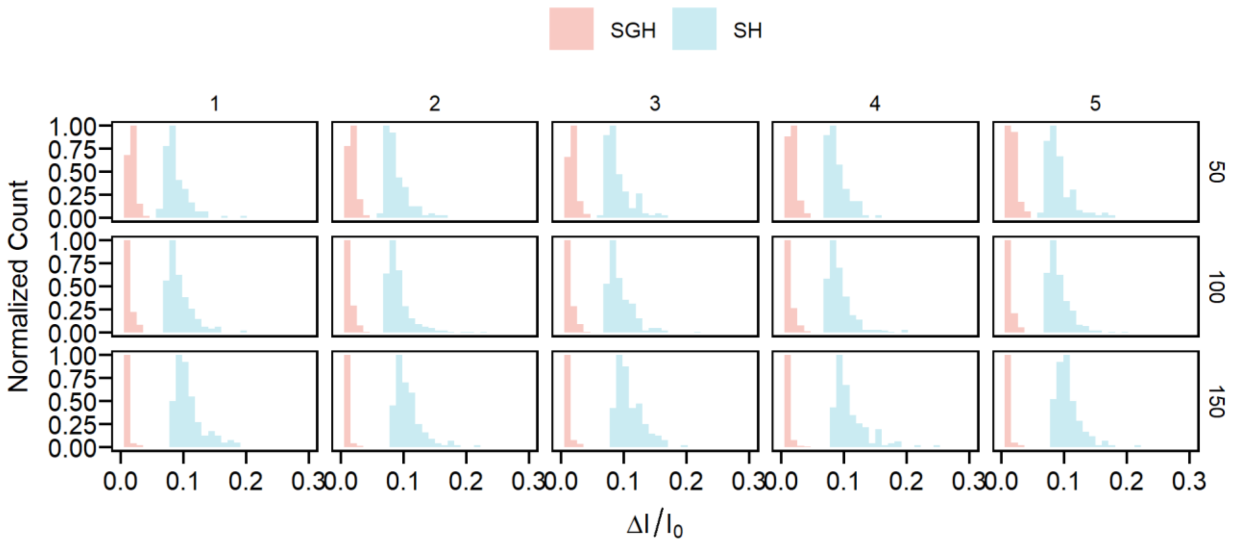


**Figure S 16 Comparative analysis of relative current drop between SGH and SH across all devices and voltages.**

The normalized distributions of relative current blockade ($\Delta I/I_0$) for SGH ($SiN_x$–graphene–hBN) and SH ($SiN_x$–hBN) nanopores under applied voltages of 50, 100, and 150 mV across five replicate devices. In all cases, SH pores display broader blockade distributions with peaks that extend toward higher values, typically reaching 0.2–0.25, whereas SGH pores exhibit narrower and more sharply defined distributions centered at lower values around 0.05–0.1. With increasing bias voltage, both SH and SGH distributions don't shift too much. Importantly, the insertion of a graphene layer in SGH consistently reduces the average blockade depth and narrows the spread of the distribution compared to SH, indicating that graphene alters the pore–protein interaction, modulates the inside electrical field distribution and results in smaller translocation signals.

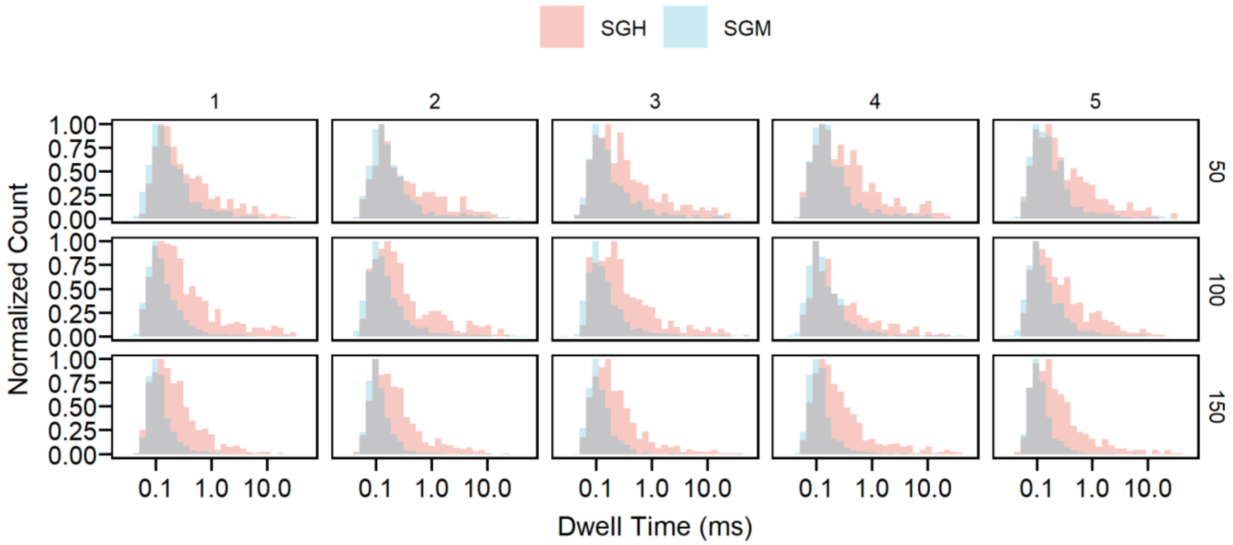


**Figure S 17 Comparative analysis of dwell time between SGH and SGM across all devices and voltages.**

This figure presents the normalized dwell time distributions for SGH and SGM nanopores under voltages of 50, 100, and 150 mV across five replicate devices. At all applied biases, SGM pores consistently yield shorter dwell times, with distributions peaking below 1 ms and decaying rapidly, whereas SGH pores display broader and more extended tails reaching up to tens of milliseconds. The difference becomes especially apparent at higher voltages, where SGM events shift toward even shorter dwell times, while SGH events retain a considerable fraction of long translocations. The comparison across replicate devices shows the consistency of the CT-CDB fabrication.

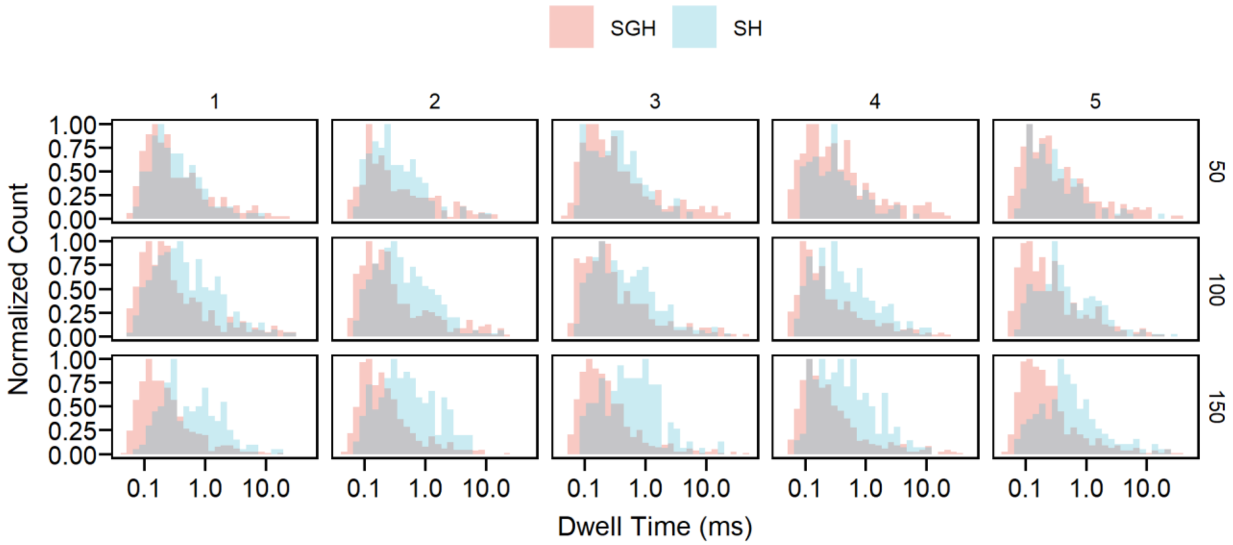


**Figure S 18 Comparative analysis of dwell time between SGH and SH across all devices and voltages.** This figure shows the normalized dwell time distributions for SGH and SH nanopores measured at 50, 100, and 150 mV across five replicate devices. In general, SGH pores exhibit shorter dwell times compared to SH, with distributions shifted toward sub-millisecond events and reduced contributions from long translocations. SH pores, on the other hand, consistently display broader distributions with heavier tails extending into several milliseconds. As the applied voltage increases, SGH distributions shift toward shorter dwell times, reflecting the expected acceleration of translocation under stronger electric fields, but the relative difference between the two structures persists. These results suggest that the insertion of a graphene layer in SGH reduces effective pore length, thereby producing faster translocation kinetics compared to SH nanopores.

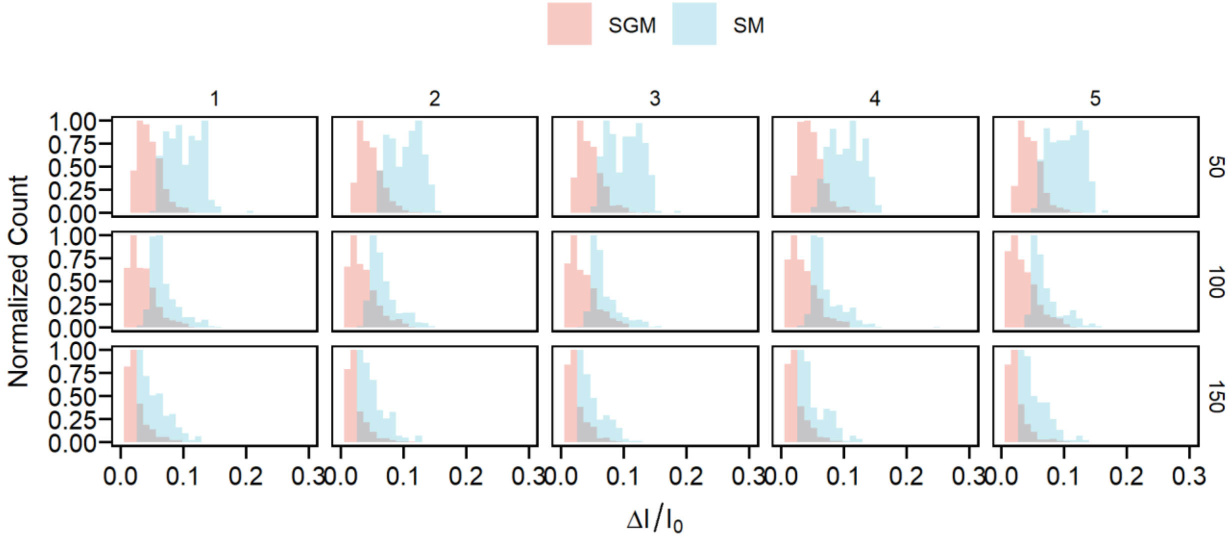


**Figure S 19 Comparative analysis of relative current drop between SGM and SM across all devices and voltages.**

This figure compares the normalized histograms of relative current blockade ($\Delta I/I_0$) for SGMand SM nanopores under applied voltages of 50, 100, and 150 mV across five replicate devices. In general, SM pores exhibit broader distributions that extend to higher blockade values, often exceeding 0.15–0.20, while SGM pores show narrower distributions centered at lower $\Delta I/I_0$, typically below 0.1. The insertion of graphene in SGM thus reduces the overall blockade depth and narrows the spread, suggesting weaker protein–pore interactions and more uniform current modulation compared to SM. As voltage increases, both structures show a gradual shift toward lower blockade values, consistent with the reduced relative current drop under stronger driving fields. These results indicate that graphene mitigates the strong interaction effects introduced by $MoS_2$, leading to smaller and more stable current blockades in multilayer nanopore architectures.

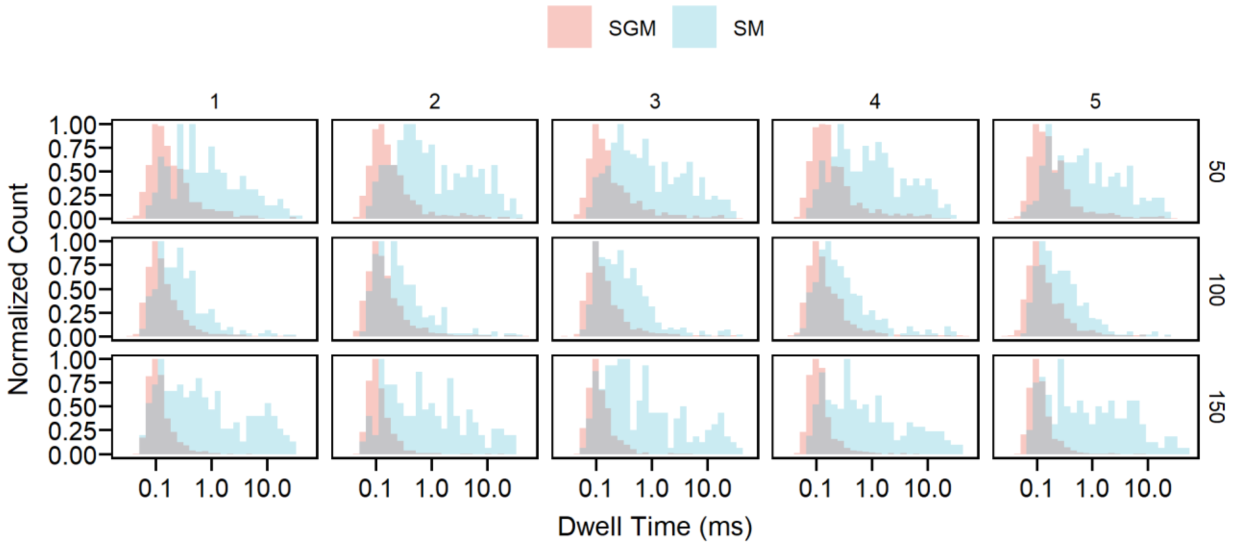


**Figure S 20 Comparative analysis of dwell time between SGM and SM across all devices and voltages.**

The normalized dwell time distributions for SGM and SM nanopores are compared under applied voltages of 50, 100, and 150 mV across five replicate devices. SM pores display broader dwell time distributions with long tails extending into the 10–100 ms range, indicating the frequent occurrence of longer translocation events. In contrast, SGM pores show narrower distributions with peaks concentrated at shorter timescales, typically below 1 ms, and significantly fewer longer dwell events. With increasing voltage, both structures exhibit the slight shift toward shorter dwell times, reflecting faster molecular passage under stronger electric fields, but the relative difference remains robust: SM continues to display longer and more variable events, while SGM maintains faster and more uniform kinetics. These findings suggest that graphene insertion into $MoS_2$-based pores reduces strong molecule–surface interactions and suppresses trapping effects, thereby shortening dwell times.

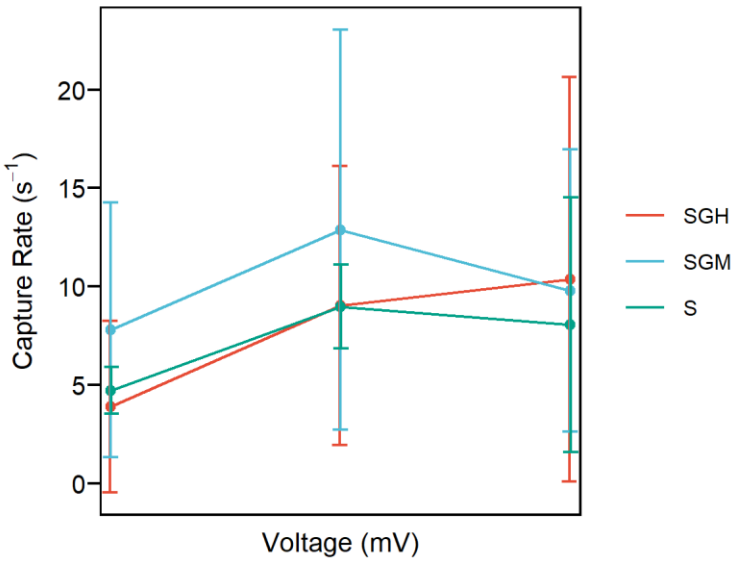


**Figure S 21 Capture rate comparison among tri-layer nanopores and bare $SiN_x$ nanopores.**

All of the capture rate variations are within the error margin while at 100 mV and 150 mV, tri-layer structures have slightly higher capture rate. No apparent degradation appears with tri-layer structures.

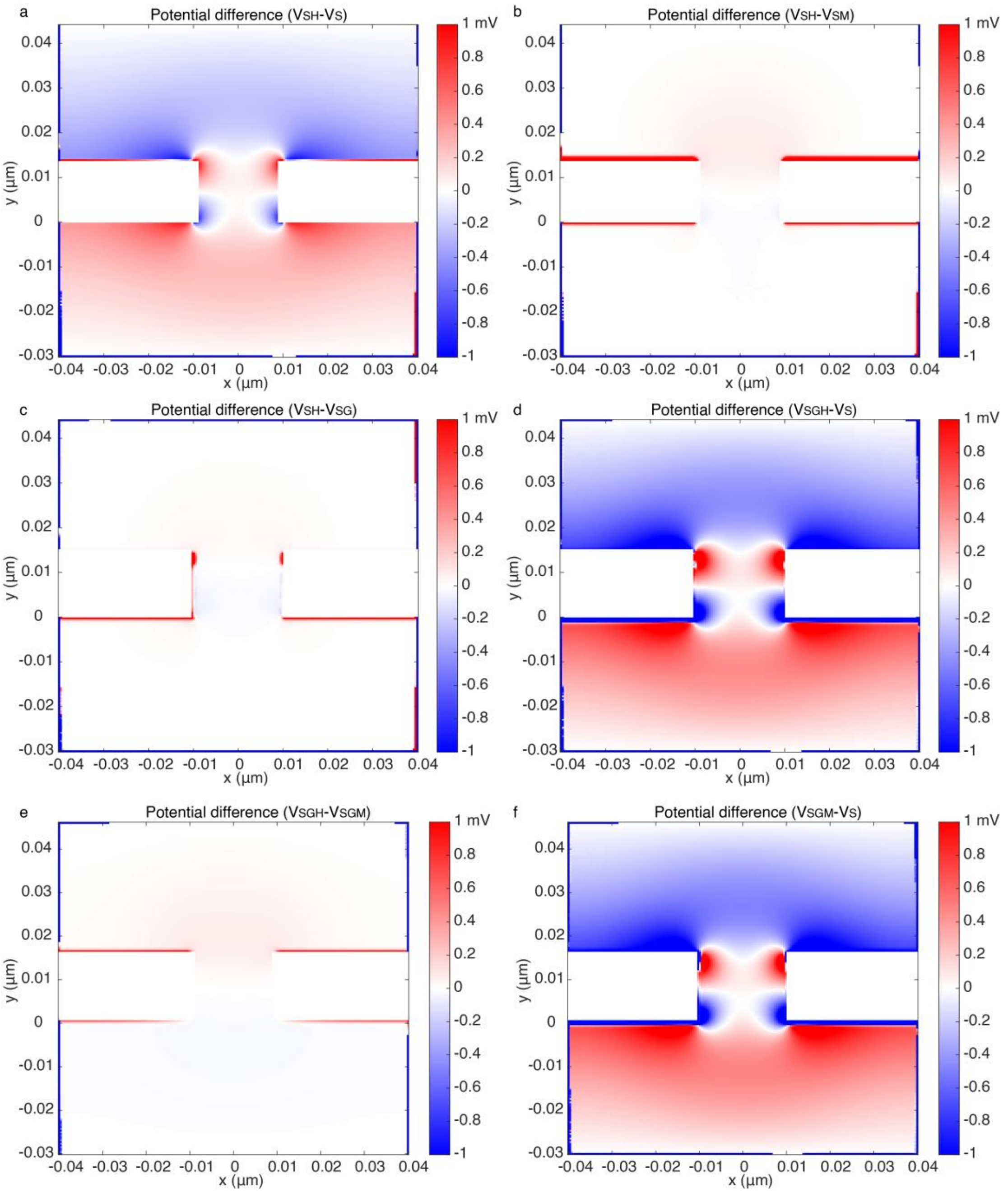


**Figure S 22 COMSOL simulation for electric potential difference among various nanopore structures at 100 mV.**

COMSOL Multiphysics simulations of electric potential profiles for various nanopore membrane structures, each subjected to 100 mV transmembrane bias. Each subplot corresponds to a specific membrane configuration, as indicated in the titles. Differences in the electric field gradient and potential drop across the pore are evident among the architectures, with multilayer structures exhibiting attenuated and spatially distributed field profiles compared to bare $SiN_x$. These variations influence local ionic environment and protein–pore interaction strength, contributing to differences in translocation behavior observed experimentally.

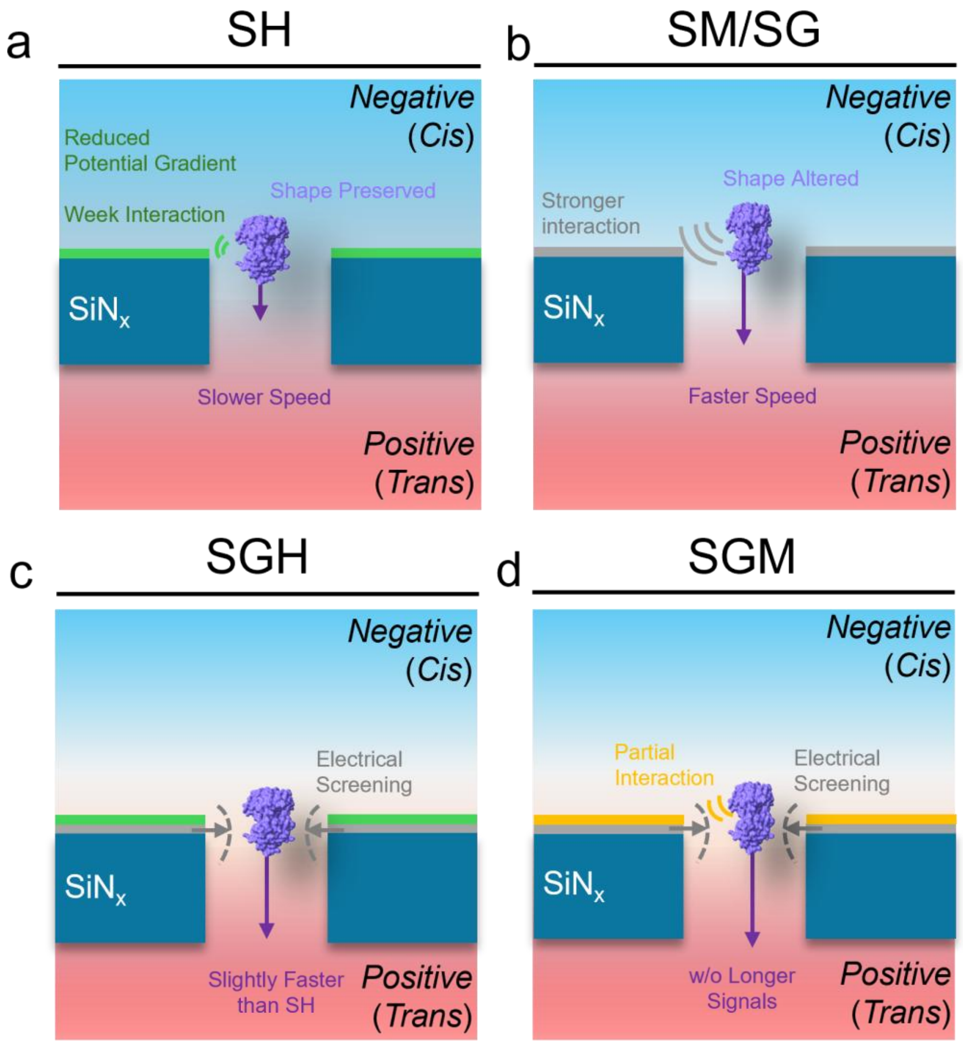


**Figure S 23 Diagram of the mechanism governing biomolecule translocation in multilayer nanopores.**

This figure illustrates the key effects influencing protein translocation in multilayer nanopore structures with different material combinations. Each subplot depicts the characteristic structure-translocation features for a specific architecture: (a) SH, with the green layer representing hBN; (b) SM/SG, with the gray layer representing $MoS_2$ or Graphene; (c) SGH, with the gray layer representing graphene and the green layer representing hBN; and (d) SGM, with the gray layer representing graphene and the yellow layer representing $MoS_2$. Owing to the distinct physicochemical properties of the constituent materials, the strength of protein-pore interactions varies among the different multilayer structures.

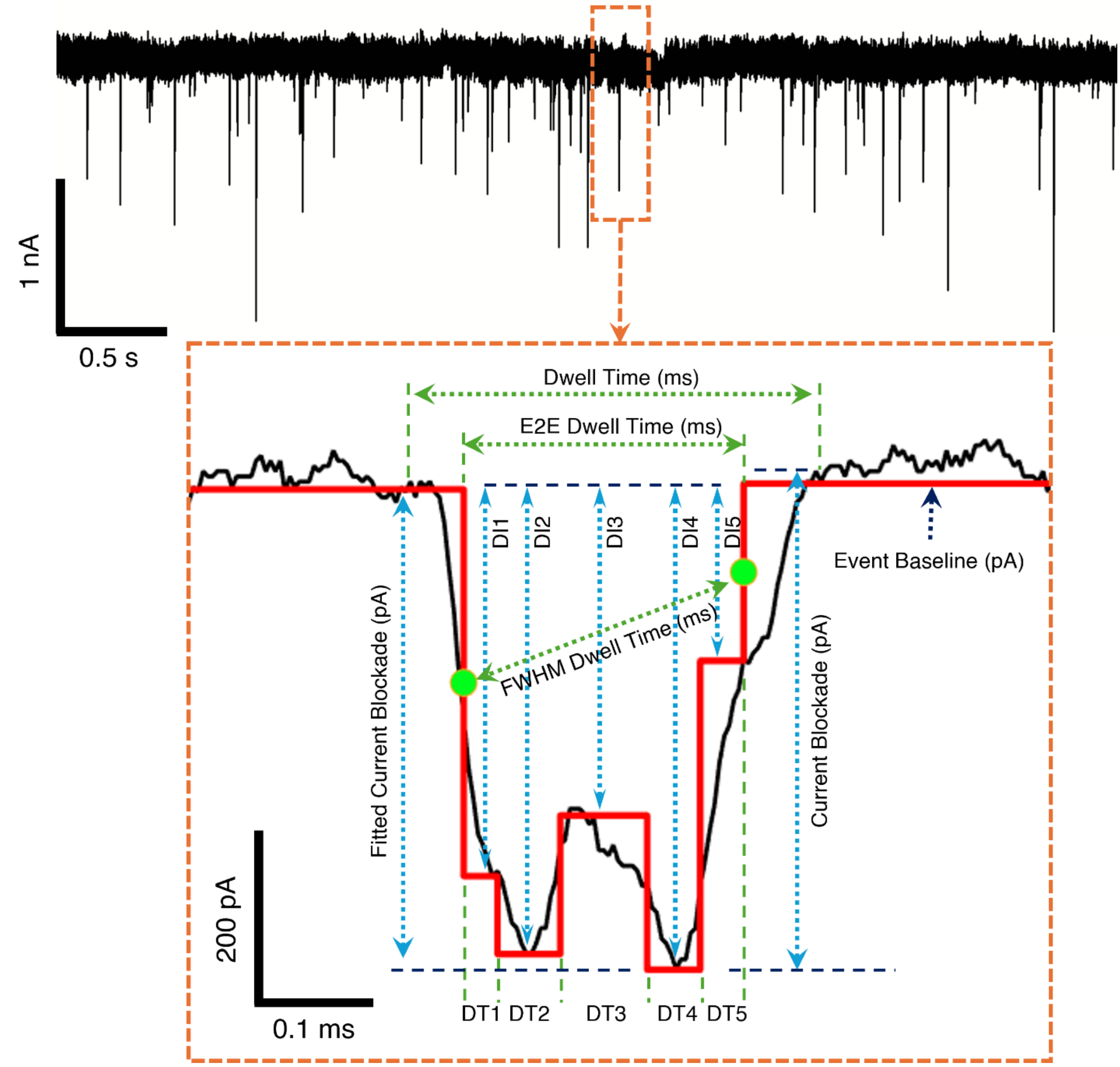


**Figure S 24 Annotated sample ionic current trace with extracted features.**

A representative raw translocation event is showed in this figure with labels corresponding to key extracted features used in the machine learning model. Parameters such as dwell time, current blockade, event baseline, and full-width at half maximum (FWHM) are annotated. This figure serves to illustrate the underlying data structure and rationale behind feature engineering.

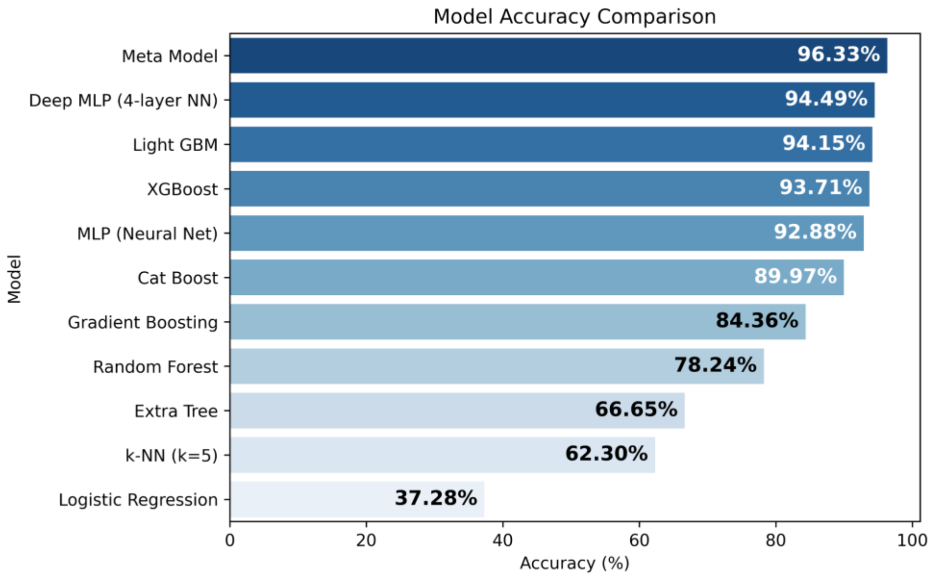


**Figure S 25 Accuracy comparison of individual classifiers.**

This figure compares the classification performance of several single models (e.g., MLP, ExtraTrees, LightGBM, XGBoost) when trained and evaluated on the same dataset. Accuracy is used as the primary metric. While tree-based models showed strong performance, their variance across folds justified the ensemble stacking approach used in the main manuscript.

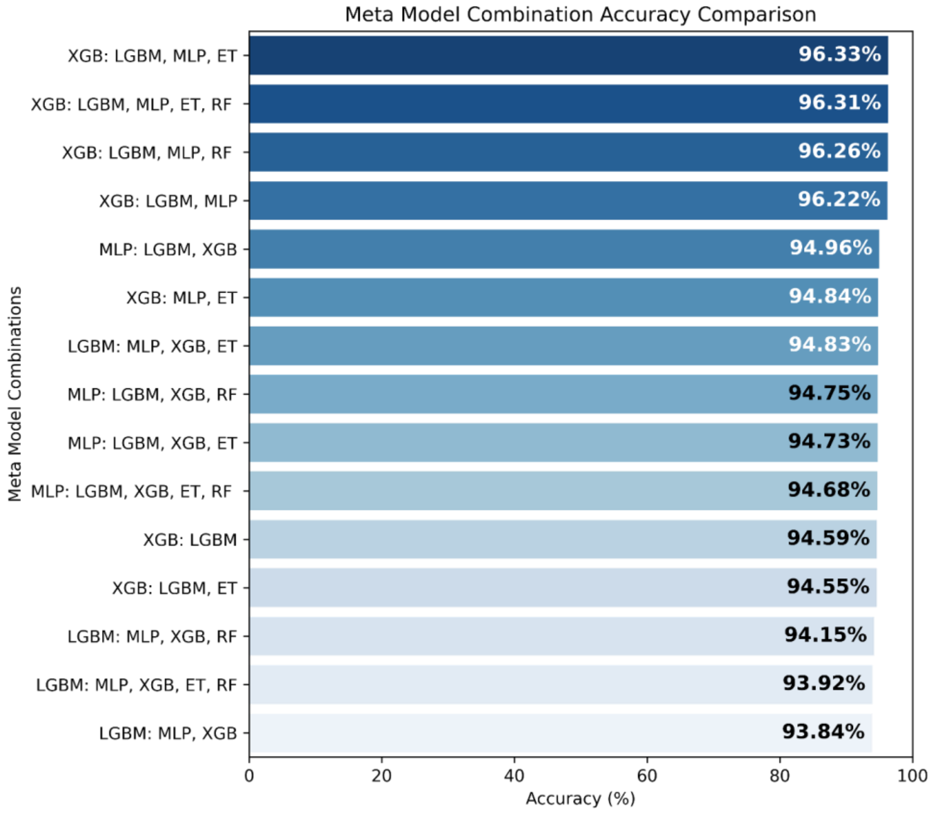


**Figure S 26 Evaluation of stacked model variants using different base learner combinations.**

To optimize the final ensemble structure, different combinations of base learners were benchmarked. This figure shows classification accuracy for each tested combination, confirming that inclusion of both tree-based and neural models enhances generalization compared to single-model baselines.

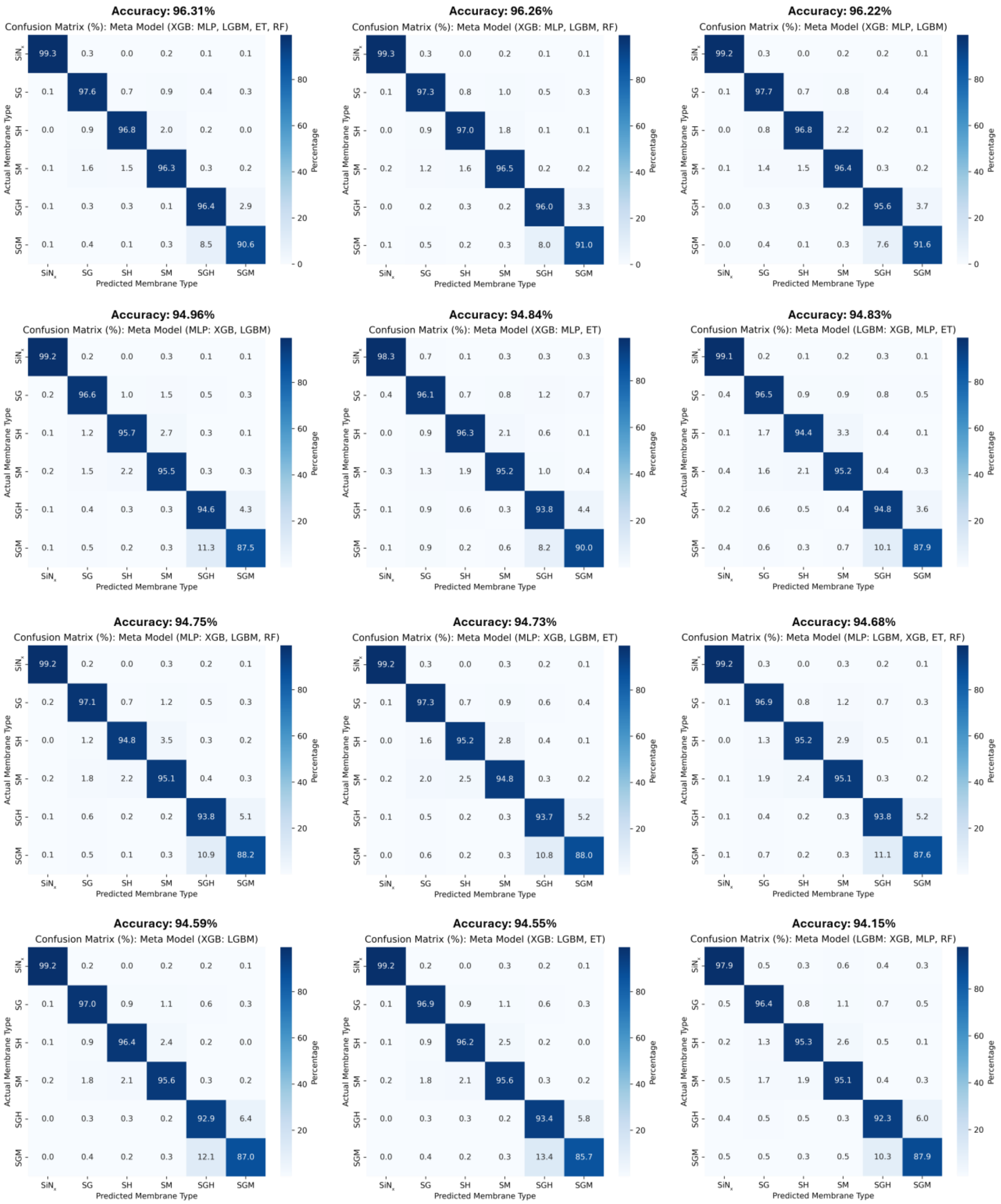


**Figure S 27 Accuracy ranking of the top 12 (excluding final model) stacked meta-model configurations.**

In this figure, each model represents a different ensemble configuration with varying base learner pairings and meta-learners. Models were trained and evaluated using the same cross-validation strategy. The final model used in the main analysis (Figure 5) achieved the highest accuracy of 96.33%.

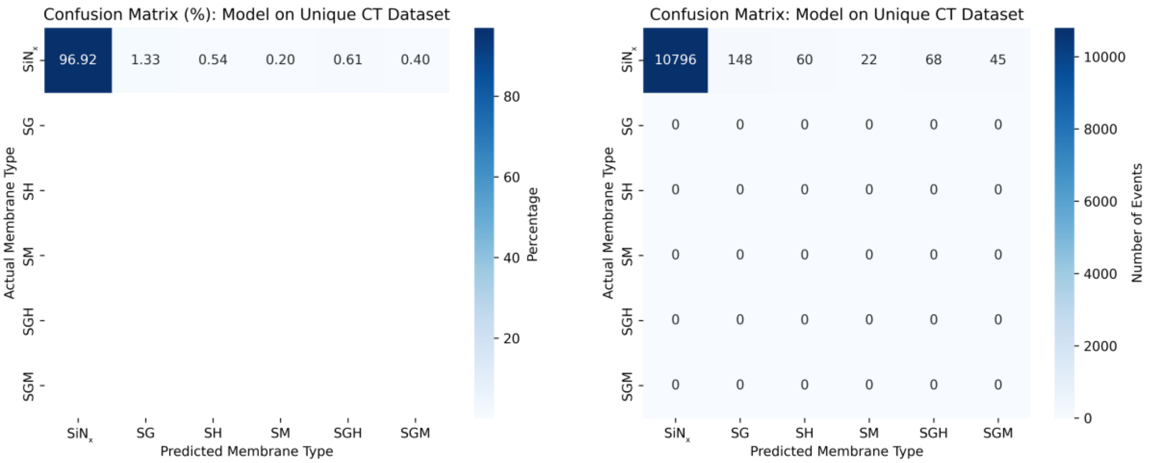


**Figure S 28 Confusion matrix of the stacked model evaluated on a unique $SiN_x$-only dataset.**

The performance of the final stacked meta-model was evaluated on a distinct dataset composed entirely of events from bare $SiN_x$ nanopores, recorded under similar voltage and buffer conditions. The resulting confusion matrix shows accurate classification of membrane-type subpopulations within the $SiN_x$ category, achieving a test accuracy of 96.62%. This confirms the model's robustness in detecting subtle structural signatures within nominally homogeneous systems.

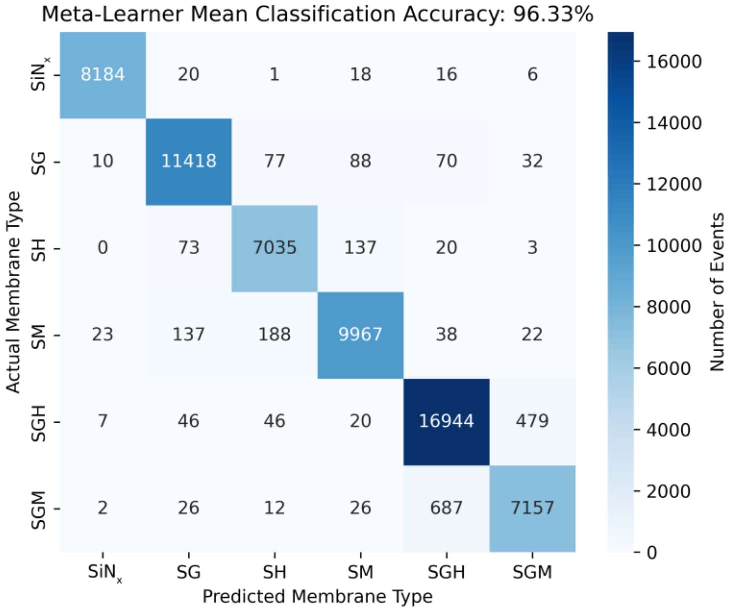


**Figure S 29 Confusion matrix showing absolute event counts classified by the ensemble model.**

Confusion matrix presenting absolute event numbers from the test dataset classified by the stacked ensemble learning model. The matrix confirms high predictive reliability and accurate differentiation among six membrane types: bare $SiN_x$, $SiN_x$+graphene (SG), $SiN_x$+$MoS_2$ (SM), $SiN_x$+hBN (SH), $SiN_x$+graphene+$MoS_2$ (SGM), and $SiN_x$+graphene+hBN (SGH). Correct predictions dominate the diagonal entries, indicating strong model performance and clear membrane-type separation.

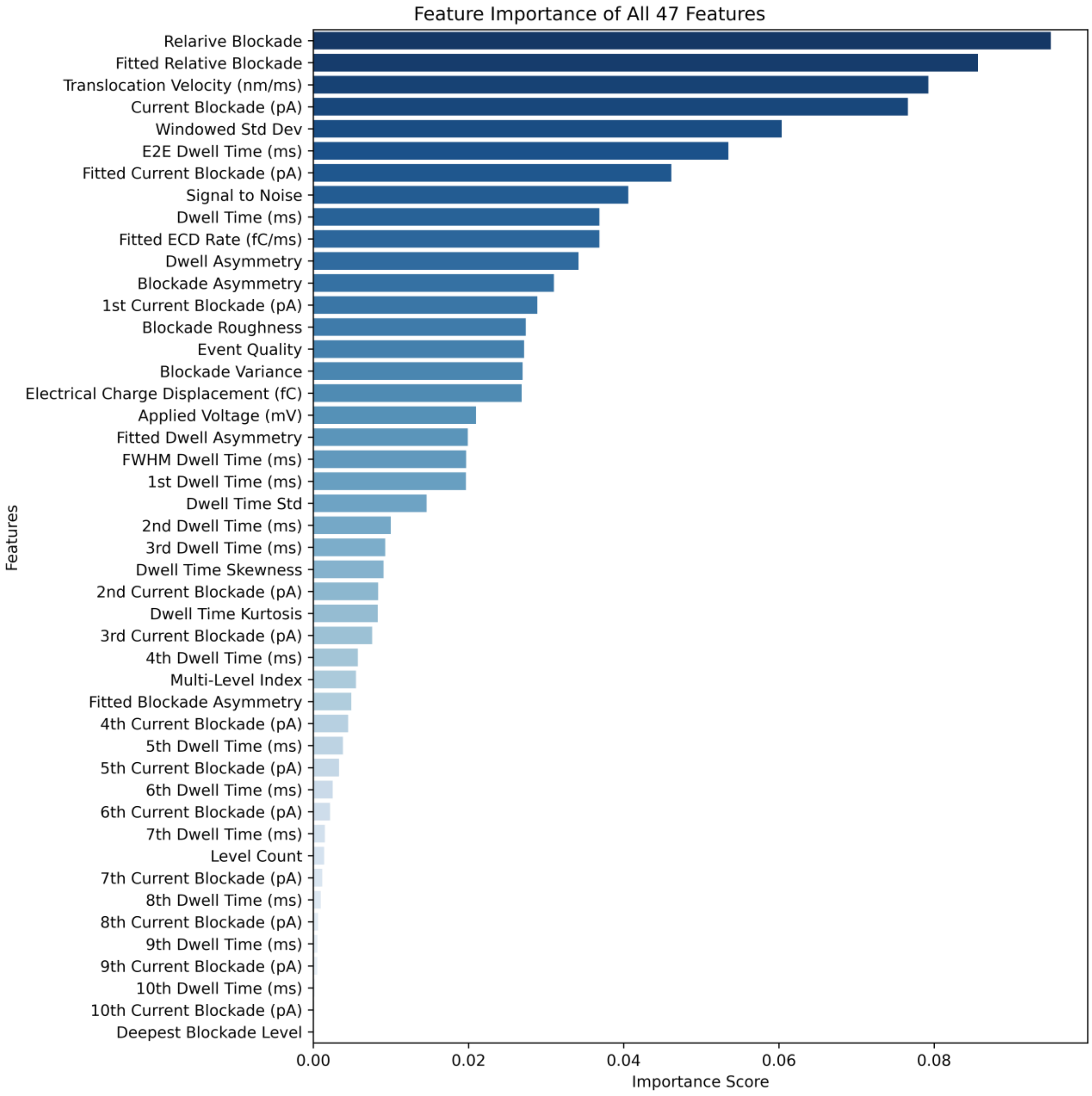


**Figure S 30 Importance scores of all 47 features utilized for classification.**

This feature ranking plot displays the relative contribution of each feature to model predictions, as inferred from a high-performing gradient-boosted model. Features such as relative blockade, velocity, ECD rate, and dwell asymmetry were consistently ranked highest, suggesting that both geometric and dynamic properties of translocation events play key roles in discriminating membrane types.

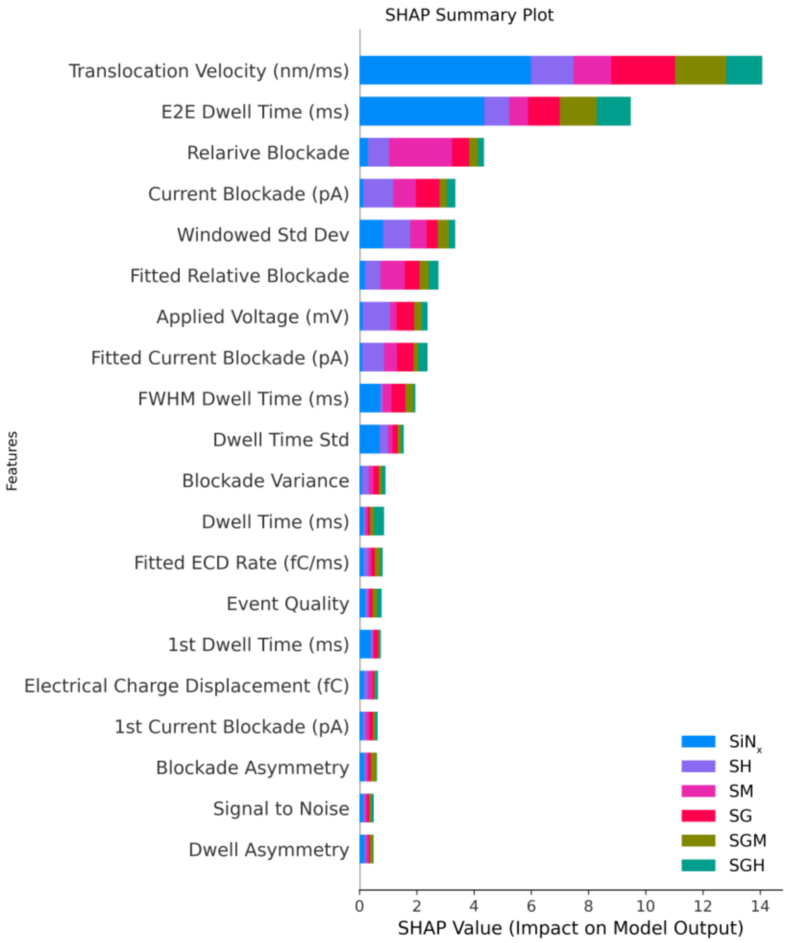


**Figure S 31 SHAP (SHapley Additive exPlanations) summary plot for model interpretability.**

SHAP values quantify the marginal contribution of each feature to individual predictions across the test set. This figure shows global feature importance and individual event variability, highlighting the predictive influence of features such as fitted relative blockade, signal-to-noise ratio, and dwell time across all membrane classes.

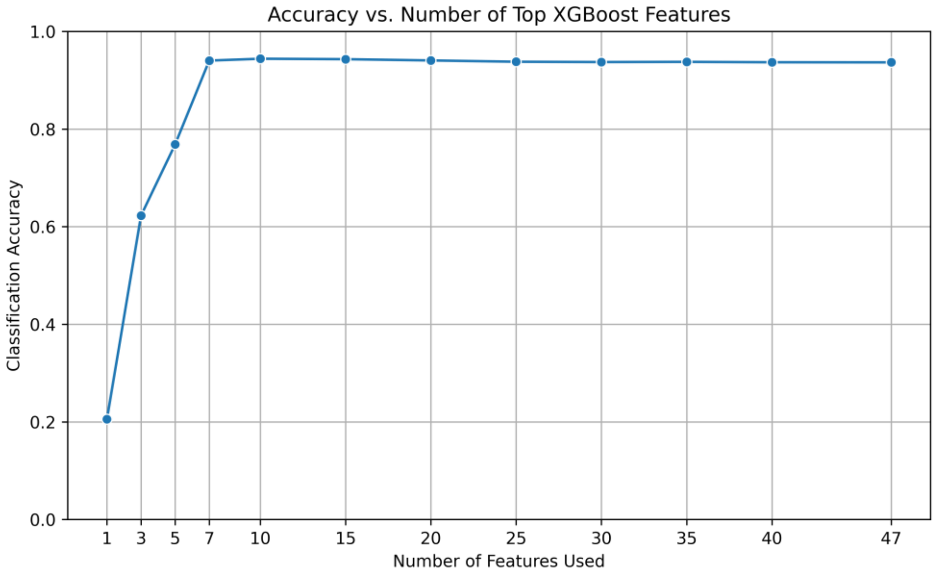


**Figure S 32 Classification accuracy of XGBoost versus number of top-ranked features used.**

XGBoost classifiers were trained using increasing subsets of the most important features (as ranked by the model itself). This figure shows how classification accuracy changes as feature count increases, plateauing after approximately 10–12 features, indicating model sparsity and effective feature selection.

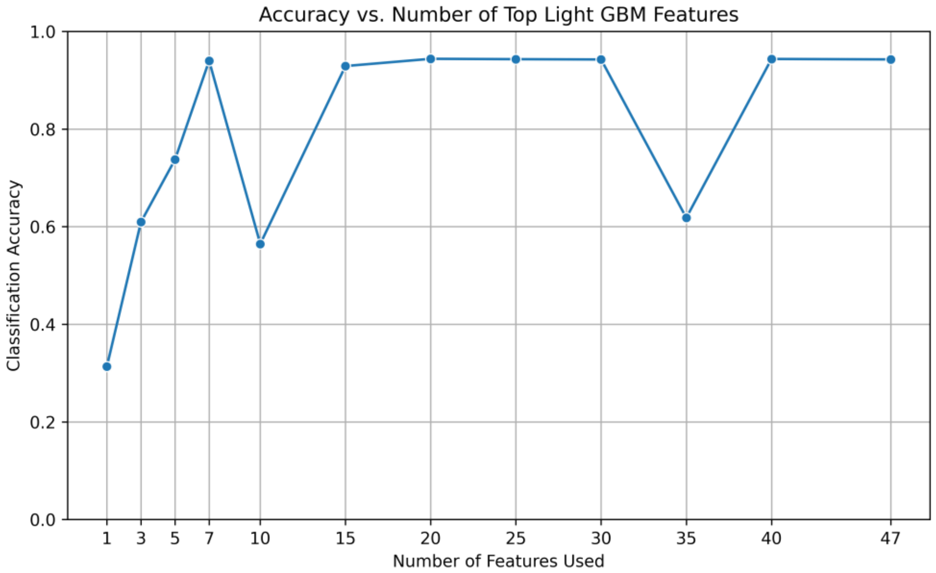


**Figure S 33Classification accuracy of LightGBM as a function of the number of top-ranked features.**

Membrane classification accuracy was evaluated using LightGBM models trained on incrementally increasing subsets of the top-ranked features. While the overall trend mirrors that observed in Figure S29 for XGBoost, showing near-maximal accuracy with approximately 10–15 features, LightGBM exhibits noticeable accuracy dips around feature counts of ~10 and ~35. This deviation may arise from LightGBM's leaf-wise tree growth strategy, which can lead to local overfitting or suboptimal feature splits when the input space is reduced or non-uniformly scaled. In contrast, XGBoost's depth-wise approach appears more robust under these conditions. These results highlight the sensitivity of different gradient boosting algorithms to feature selection strategies and reinforce the importance of model-specific optimization in stacked ensemble frameworks.

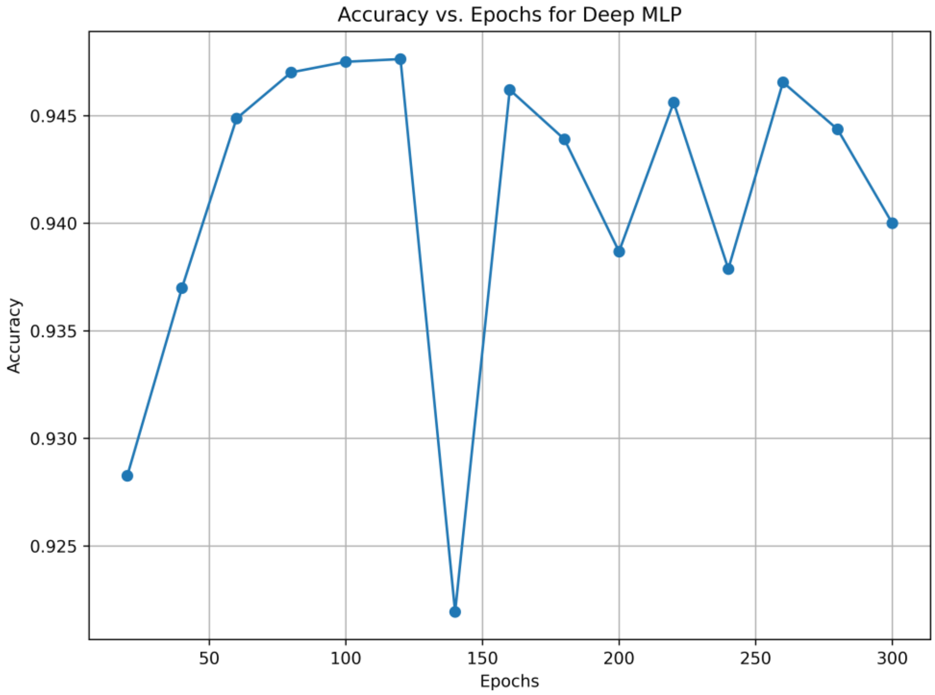


**Figure S 34 MLP classifier accuracy versus number of training epochs.**

A learning curve plot showing the classification accuracy of the MLP model over increasing training epochs. Accuracy increases steadily before reaching a plateau, supporting the selection of 120 epochs as an optimal balance between performance and computational cost.

## Supplementary Notes

Volume Exclusion Model Note

To quantify the ionic current blockades produced by holo-transferrin translocating through a multilayer nanopore, we employed the classical volume exclusion model, in which the relative current drop is approximated by the fraction of the effective pore volume displaced by the molecule. The nanopore was modeled with a nominal cylindrical geometry of 20 nm diameter ($r = 10\, nm$) and 14 nm thickness, immersed in 1 M KCl ($\sigma \approx 11.5\, S \cdot m^{-1}$). The effective pore volume is therefore $\pi r^2 L \approx 4398\, nm^3$.

Treating holo-transferrin as a sphere of effective diameter ~8 nm[13] ($V_{hSTf} \approx 268\, nm^3$), the relative current blockade predicted by the volume exclusion model is[14]

$$\frac{\Delta I}{I_0} \approx \frac{V_{hSTf}}{V_{pore}} \approx 6.1\% \qquad (S1)$$

which is close to the $\Delta I/I_0$ distribution in SH structure (Figure 3f) across all voltages.

While the cylindrical approximation provides a convenient baseline, real nanopores often deviate from ideal geometries. For conical frusta or hourglass-like pores with end radii of 9–11 nm, the effective pore volume changes only marginally, resulting in blockade ratios very close to the cylindrical case (~6.0%). However, resistance-weighted formulations that account for local current density distributions emphasize narrow waists or entrances, leading to slightly higher blockade fractions (typically +2–5% relative to the volume-ratio model). Similarly, elliptical cross-sections with the same projected area preserve the predicted blockade fraction, although a reduced minor axis at the entrance or waist again increases the effective blockade due to current focusing.

These results suggest that an 8 nm transferrin molecule should induce a blockade of ~6% in a 20 nm × 14 nm nanopore in 1 M KCl. Deviations from perfect cylindrical geometry, particularly constrictions or waist-like features, are expected to increase the blockade modestly away from the volume ratio prediction. These calculations provide a realistic first-order baseline for interpreting experimental translocation data in multilayer solid-state nanopores.

COMSOL Simulation Note

Considering the different conducting types for various structures, it's meaningful to simulate the electric potential distribution for different nanopores and see what insights these possible differences can provide to us concerning the specific experimental results.

We use 2D geometry model. The thicknesses of $SiN_x$ and extra layer are 12 nm and 2 nm for simplification. Pore diameter is 20 nm. Two reservoir regions have same dimension as 80 nm × 30 nm.

We use two basic physics modules for our simulation: *Electrostatics* and *Transport of Diluted Species*. Since we use 1M KCl solution, the concentration of electrolyte is high enough and we can ignore the effect of electric double layer (Debye Length < 0.3 nm)[15]. Therefore, the system is dominated by electrophoresis. Based on this, we don't introduce the fluid module into our simulation.

The simulations assume continuum electrostatics and steady-state ionic transport, which are standard approximations for the nanopores. Main boundary conditions include: (i) *Electrostatics:* charge conservation on whole structure, zero charge at the outer edge, zero space charge density inside material, specific surface charge density on the surface and the edge inside pore (see values in Table S1); (ii) *Transport of Diluted Species:* convection and "migration in electric field" are included, electrolyte species ±1 for $K^+$and $Cl^-$, initial value of electrolyte concentration 1 M, and initial inflow from top and bottom reservoir edges.

Three typical external voltage values are set: 50 mV, 100 mV and 150 mV. By using Poisson equation [equation (S2)] and Nernst-Planck equation [equation (S3)]

$$\nabla^2\psi = -\frac{F}{\epsilon_0\epsilon_r}\sum_{i=1}^{2} z_i\boldsymbol{c}_i \tag{S2}$$

$$\mathbf{J}_i = -D_i\nabla\boldsymbol{c}_i - \frac{z_iF}{RT}D_i\boldsymbol{c}_i\nabla\boldsymbol{\Phi} \pm \mathbf{v}_c\mathbf{c}_i \tag{S3}$$

the electric field and the migration of ions are coupled together. In these equations, $\psi$ is the electric potential, $F$ is the Faraday constant, $\epsilon_0$ the dielectric constant in vacuum, $\epsilon_r$ the relative dielectric constant, $z_i$ the valency of ion $i$, $\boldsymbol{c}_i$ the ions molar concentration, $\mathbf{J}_i$ the total flux per area of ion $i$, $D_i$ the diffusion coefficient, $R$ the gas constant, $T$ the temperature, $\boldsymbol{\Phi}$ the electric potential, and $\mathbf{v}_c$ the convective velocity field.

Figure S21 shows the potential difference among various nanopore structures at 100 mV. Different voltage only influences the potential quantity, not the distribution. In general, for single nanopore device, no matter what structure it is, the potential drop is concentrated to pore area. However, some minor differences can actually affect the behavior of protein translocation. In Figure S21a, the potential difference between SH and $SiN_x$ has complex change in pore area. For hSTf negatively charged in our electrolyte, the translocation direction is from top to bottom in the figure. At the pore entrance, the potential of SH becomes higher than $SiN_x$, and for hSTf, it can easily go inside the channel. At the exit part, potential goes lower, which hinders the translocation. This inner change

causes unstable movement of the molecules, and the translocation speed is slowed down. Similar situation happens in SGH (Figure S21d) and SGM (Figure S21f) structures. The comparison among bilayer structures and multilayer structures shows another mode. From Figure S21b, c, e, we notice that nanopore structure with hBN layer has more gradual potential gradient change, which leads to relatively weaker electrophoretic force according to $F = -q\nabla\phi$ and hence slower translocation speed for hSTf proteins. Reflected in the statistical analysis in main text (Figure 3 and Figure 4), more events have longer dwell time. The speed, or velocity also exhibits its significance from machine learning, which turns out to be the top three most important features influenced by various nanopore structures.

## Machine Learning Note

To classify nanopore membrane types based on translocation event signatures, we implemented a supervised machine learning pipeline comprising data preprocessing, feature engineering, model training, and performance evaluation.

### Feature extraction and preprocessing

Translocation events were identified from ionic current traces and parameterized using engineered features derived from baseline-normalized signal segments. Each event was represented by a multi-dimensional feature vector encompassing current blockade ($\Delta I$), dwell time ($\Delta t$), relative blockade ($\Delta I/I_0$), signal-to-noise ratio (SNR), event area (ECD), event asymmetry, translocation velocity, and statistical descriptors such as skewness and kurtosis. These features were z-score normalized before model input. Events were randomly split into training and test datasets with stratified class representation. Missing or undefined values were excluded from analysis.

### Stacked ensemble model architecture

We employed a stacked ensemble learning strategy to enhance classification performance across the six membrane classes. The base learners included a multilayer perceptron (MLP) implemented in PyTorch, an Extremely Randomized Trees classifier, and a Light Gradient Boosted Machine (LightGBM) model. The MLP consisted of fully connected layers with ReLU activation and was trained using the Adam optimizer and cross-entropy loss for 120 epochs with a batch size of 1024. The meta-learner was implemented using XGBoost with a maximum tree depth of 10 and 500 boosting rounds. Predictions from the base models were passed to the meta-learner using a 5-fold cross-validation framework with passthrough enabled.

### Model evaluation and feature interpretation

Classification performance was evaluated on a held-out test set using accuracy and confusion matrices. Feature importance was assessed using built-in scoring metrics from tree-based models. To provide model interpretability, SHAP (SHapley Additive exPlanations) values were computed for the best-performing classifier, quantifying the contribution of each feature to model predictions. To assess model sparsity and performance stability, classification accuracy was evaluated as a function of the number of top-ranked features.

### Software and hardware

Model training and evaluation were performed using Python (v3.10), PyTorch, scikit-learn, XGBoost, LightGBM, and SHAP libraries. Training was conducted on a workstation equipped with an NVIDIA RTX 4060 GPU, 64 GB RAM, and an Intel i7-14700F processor.

## Reference


(1) Laturia, A.; Van de Put, M. L.; Vandenberghe, W. G. Dielectric Properties of Hexagonal Boron Nitride and Transition Metal Dichalcogenides: From Monolayer to Bulk. *npj 2D Mater Appl* **2018**, *2* (1), 6. https://doi.org/10.1038/s41699-018-0050-x.

(2) Stauber, T.; Peres, N. M. R.; Geim, A. K. Optical Conductivity of Graphene in the Visible Region of the Spectrum. *Phys. Rev. B* **2008**, *78* (8), 085432. https://doi.org/10.1103/PhysRevB.78.085432.

(3) Smeets, R. M. M.; Keyser, U. F.; Krapf, D.; Wu, M.-Y.; Dekker, N. H.; Dekker, C. Salt Dependence of Ion Transport and DNA Translocation through Solid-State Nanopores. *Nano Lett.* **2006**, *6* (1), 89–95. https://doi.org/10.1021/nl052107w.

(4) Vlassiouk, I.; Smirnov, S.; Siwy, Z. Nanofluidic Ionic Diodes. Comparison of Analytical and Numerical Solutions. *ACS Nano* **2008**, *2* (8), 1589–1602. https://doi.org/10.1021/nn800306u.

(5) Garaj, S.; Hubbard, W.; Reina, A.; Kong, J.; Branton, D.; Golovchenko, J. A. Graphene as a Subnanometre Trans-Electrode Membrane. *Nature* **2010**, *467* (7312), 190–193. https://doi.org/10.1038/nature09379.

(6) Feng, J.; Liu, K.; Graf, M.; Dumcenco, D.; Kis, A.; Di Ventra, M.; Radenovic, A. Observation of Ionic Coulomb Blockade in Nanopores. *Nature Mater* **2016**, *15* (8), 850–855. https://doi.org/10.1038/nmat4607.

(7) Gholamy, A.; Kreinovich, V.; Kosheleva, O. Why 70/30 or 80/20 Relation Between Training and Testing Sets: A Pedagogical Explanation. *Departmental Technical Reports (CS)* **2018**.

(8) Gorbachev, R. V.; Riaz, I.; Nair, R. R.; Jalil, R.; Britnell, L.; Belle, B. D.; Hill, E. W.; Novoselov, K. S.; Watanabe, K.; Taniguchi, T.; Geim, A. K.; Blake, P. Hunting for Monolayer Boron Nitride: Optical and Raman Signatures. *Small* **2011**, *7* (4), 465–468. https://doi.org/10.1002/smll.201001628.

(9) Ferrari, A. C.; Basko, D. M. Raman Spectroscopy as a Versatile Tool for Studying the Properties of Graphene. *Nature Nanotech* **2013**, *8* (4), 235–246. https://doi.org/10.1038/nnano.2013.46.

(10) Lee, C.; Yan, H.; Brus, L. E.; Heinz, T. F.; Hone, J.; Ryu, S. Anomalous Lattice Vibrations of Single- and Few-Layer MoS2. *ACS Nano* **2010**, *4* (5), 2695–2700. https://doi.org/10.1021/nn1003937.

(11) Carvalho, B. R.; Wang, Y.; Mignuzzi, S.; Roy, D.; Terrones, M.; Fantini, C.; Crespi, V. H.; Malard, L. M.; Pimenta, M. A. Intervalley Scattering by Acoustic Phonons in Two-Dimensional MoS2 Revealed by Double-Resonance Raman Spectroscopy. *Nat Commun* **2017**, *8* (1), 14670. https://doi.org/10.1038/ncomms14670.

(12) Pimenta, M. A.; del Corro, E.; Carvalho, B. R.; Fantini, C.; Malard, L. M. Comparative Study of Raman Spectroscopy in Graphene and MoS2-Type Transition Metal Dichalcogenides. *Acc. Chem. Res.* **2015**, *48* (1), 41–47. https://doi.org/10.1021/ar500280m.

(13) Saharia, J.; Bandara, Y. M. N. D. Y.; Goyal, G.; Lee, J. S.; Karawdeniya, B. I.; Kim, M. J. Molecular-Level Profiling of Human Serum Transferrin Protein through Assessment of Nanopore-Based Electrical and Chemical Responsiveness. *ACS Nano* **2019**, *13* (4), 4246–4254. https://doi.org/10.1021/acsnano.8b09293.

(14) Li, J.; Fologea, D.; Rollings, R.; Ledden, B. Characterization of Protein Unfolding with Solid-State Nanopores. *PPL* **2014**, *21* (3), 256–265. https://doi.org/10.2174/09298665113209990077.

(15) Schoch, R. B.; Han, J.; Renaud, P. Transport Phenomena in Nanofluidics. *Rev. Mod. Phys.* **2008**, *80* (3), 839–883. https://doi.org/10.1103/RevModPhys.80.839.
